# How unnecessarily high abatement costs and unresolved distributional issues undermine nutrient reductions to the Baltic Sea

**DOI:** 10.1007/s13280-021-01580-4

**Published:** 2021-06-09

**Authors:** Anna Andersson, Mark V. Brady, Johanna Pohjola

**Affiliations:** 1 Department of Economics, Swedish University of Agricultural Sciences & AgriFood Economics Centre, Box 7080, 220 07 Lund, Sweden; 2 Centre for Environmental and Climate Science (CEC), Lund University, Box 188, 221 00 Lund, Sweden; 3 Finnish Environment Institute, Latokartanonkaari 11, 00790 Helsinki, Finland

**Keywords:** Baltic Sea, Cost effectiveness, Eutrophication, Fairness, Nutrients

## Abstract

This paper systematically reviews the literature on how to reduce nutrient emissions to the Baltic Sea cost-effectively and considerations for allocating these costs fairly among countries. The literature shows conclusively that the reduction targets of the Baltic Sea Action Plan (BSAP) could be achieved at considerably lower cost, if countries would cooperate to implement the least costly abatement plan. Focusing on phosphorus abatement could be prudent as the often recommended measures—wastewater treatment and wetlands—abate nitrogen too. An implication of our review is that the potential for restoring the Baltic Sea to good health is undermined by an abatement strategy that is more costly than necessary and likely to be perceived as unfair by several countries. Neither the BSAP nor the cost-effective solution meet the surveyed criteria for fairness, implying a need for side-payments.

**Supplementary Information:**

The online version contains supplementary material available at 10.1007/s13280-021-01580-4.

## Introduction

The Baltic Sea is one of the few brackish seas in the world, creating a unique marine ecosystem. However, human activity has created environmental problems that threaten the functioning of the ecosystem. One of the most pressing environmental issues is eutrophication due to past and continued excessive inputs of the nutrients nitrogen and phosphorus. Its low average depth and limited water exchange with other seas, make the Baltic Sea particularly susceptible to eutrophication because nutrients are diluted slowly (BalticSTERN and SwAM 2013a). The problem has been known for decades and the first international convention on the protection of the marine environment of the Baltic Sea, the Helsinki Convention, was signed already in 1974 (HELCOM 1974). Although improvements in water quality have been made (Reusch et al. 2018), almost the entire Baltic Sea (97%) remains eutrophic (HELCOM 2018a).

The nine littoral countries have agreed through the intergovernmental body the Helsinki Commission (HELCOM) to reach good environmental status for the sea by 2021 by signing the Baltic Sea Action Plan (BSAP) (HELCOM 2007a). The BSAP aims to improve water quality by assigning quantitative reduction targets for nutrient emissions to the various sub-basins of the Baltic Sea, and in turn to the respective countries, while to some extent leaving the specific actions to the signatories (Thorsøe et al. 2021). The reduction targets are ambitious and based on what marine scientists estimate is needed to restore the ecosystem (HELCOM 2013a). Considerable costs are associated with reducing nutrient emissions while public resources to fund abatement are scarce. Further, whether the process for allocating costs among the different countries is perceived as fair and reasonable by each is crucial for legitimacy of BSAP in national legislatures (Birnbaum et al. 2015). A cost-effectiveness analysis contributes to resolving Baltic Sea eutrophication by finding a solution that achieves the most nutrient abatement for the least cost to society, but the resultant allocation of abatement costs among countries will not necessarily be perceived as fair and hence must be dealt with in itself. Both issues could, therefore, be contributing to slow progress in achieving the BSAP targets.

The primary aim of this paper is to systematically review the literature on how to reduce nutrient emissions to the Baltic Sea cost-effectively. The focus of the review is the Baltic-wide literature that investigates total costs of achieving specific nutrient reduction targets, particularly the targets set in the BSAP. We first identify relevant studies and thereafter analyse results on the cost effectiveness of different nutrient abatement measures and abatement strategies to see if any general conclusions can be drawn. A secondary aim is to review how the studies have allocated the estimated costs of nutrient abatement between countries and discuss the division of the cost burden among countries from fairness perspectives found in the literature. In this way we hope to derive conclusions not only about the potential for lowering the costs of reducing nutrient emissions, but also the perceived fairness and hence political legitimacy of BSAP in national legislatures.

This paper contributes by reviewing a growing literature that is of utmost importance for decision makers. We hope that our results will be able to support future decision-making and contribute to efficient water quality improvements in the Baltic Sea. This paper complements earlier reviews on cost-effective nutrient abatement such as Elofsson (2008, 2010a) that focus on the Baltic Sea, Halkos and Galani (2016) who compare nutrient reduction in the Baltic and Black Seas, and Balana et al. (2011) that analyse the Water Framework Directive in Europe. As the literature on cost-effective nutrient abatement to the Baltic Sea has evolved rapidly both in terms of data and methods used since these reviews were published, particularly due to the revised BSAP and through BONUS projects, there is a need to synthesize the numerous recent studies as well as the old. We also contribute by using the systematic review method, which maximizes the transparency and reliability throughout the review process.

Our review shows that simultaneous reductions of nitrogen and phosphorus loads are recommended from a cost-effectiveness perspective. Generally, it is more costly to reach the phosphorus targets, which means that focusing on phosphorus abatement measures is often highlighted in the reviewed studies. However, achieving the phosphorus targets also contributes to achieving the nitrogen targets to a relatively large extent since the main measures used to abate phosphorus cost-effectively, improvements in wastewater treatment and wetlands, also abate nitrogen. Reduction of phosphorous fertilization is another measure often recommended for cost-effective phosphorous abatement while reducing nitrogen fertilization is the measure most often recommended for cost-effective nitrogen abatement.

Further, results from our review show that the choice of abatement strategy highly affects the total costs of reaching the nutrient reduction targets for the Baltic Sea. The current BSAP abatement strategy is overly restrictive in the sense that each country must reduce emissions by a certain amount to each basin. The literature is conclusive on this issue: if countries would cooperate and apply the least-costly abatement strategy for each basin, total abatement costs would be substantially lower. In short, nutrient abatement that is expensive and has little effect on water quality as implicit to BSAP should and could be avoided. Finally, we find that changing the abatement strategy to a more cost-effective one will likely affect the division of the cost burden between countries and hence affect the perceived fairness of the distribution of costs among countries. As neither BSAP nor cost-effective allocations of nutrient load abatement meet the surveyed criteria for fairness, there is likely a need for side payments.

The paper is organised as follows. “The Baltic Sea Action Plan reduction targets” presents a background to the Baltic Sea Action Plan and the agreed upon reduction targets. “Materials and methods” describes the method used, systematic review, and explains how it has been adapted to this specific study. “Results of the primary literature review” presents the main results of the review of the literature and focuses on how to reduce nutrient emissions in a cost-effective way. “Cost allocation between countries” analyses the division of the abatement cost burden among countries while “Fairness in relation to the allocation of costs among countries” discusses reduction targets in relation to fairness. The paper ends with a “Concluding discussion”.

## The Baltic Sea Action Plan reduction targets

The Baltic Sea countries collaborate through the Helsinki Commission (HELCOM) and the EU with the aim to restore the Baltic Sea to good health. In 1988, the HELCOM Ministers declared their intention to reduce nutrient loads (nitrogen and phosphorus) from all countries by 50% by 1995 (HELCOM 1988). As progress in improving water quality was slow, all littoral countries (Denmark, Sweden, Finland, Estonia, Latvia, Lithuania, Russia, Poland and Germany) and the EU adopted the HELCOM Baltic Sea Action Plan (BSAP) in 2007 (HELCOM 2007a). The aim of the BSAP is to reach good environmental status by 2021. To combat eutrophication, BSAP specifies maximum allowable inputs of nitrogen and phosphorus to different sea basins, as well as the reductions needed to reach the maximum allowable nutrient inputs, i.e., the reduction targets. In 2013, the BSAP was updated as new data and research had become available (HELCOM 2013b).

**Fig. 1:**
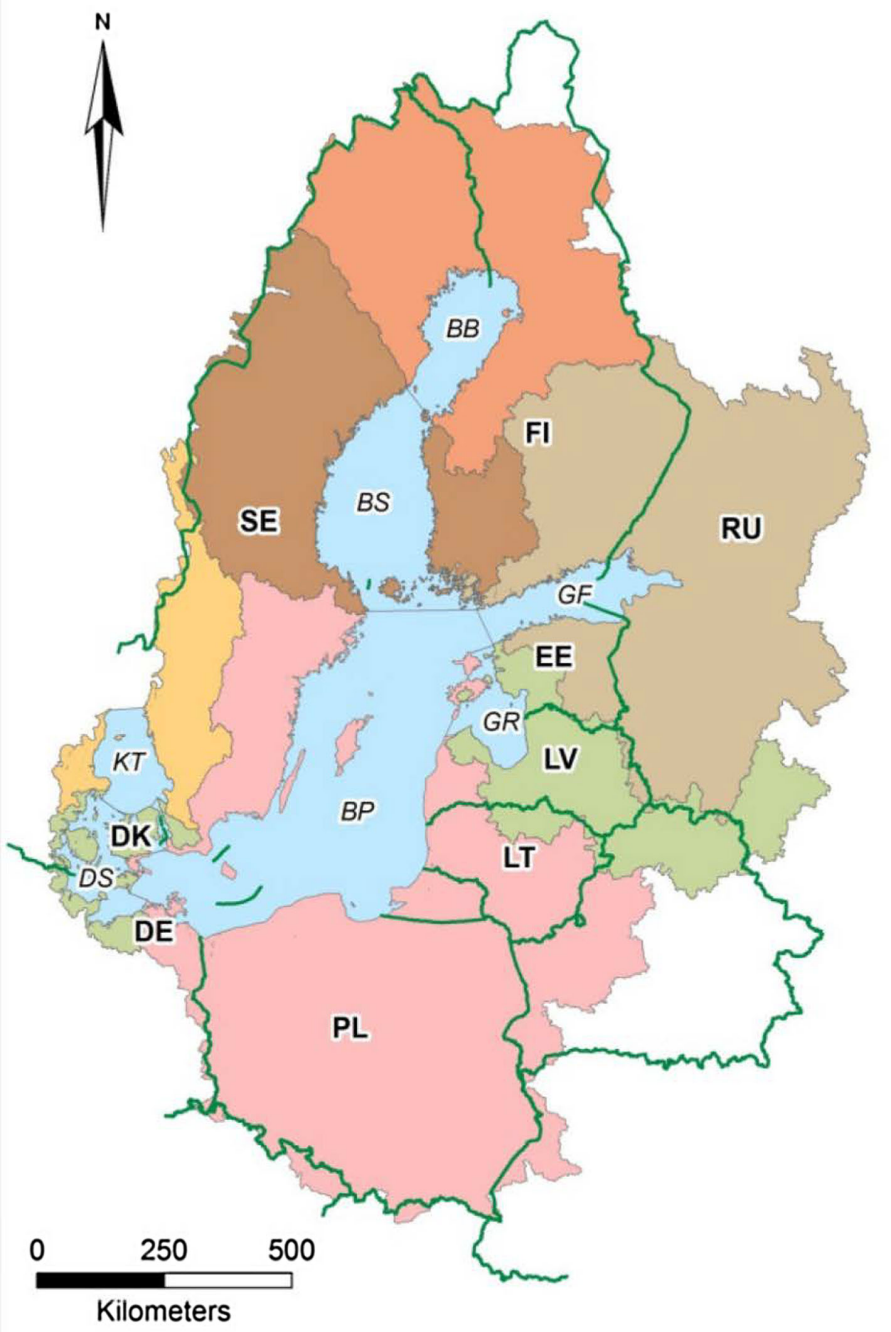
Map of the Baltic Sea basins. The different colours represent the parts of the catchment contributing to the waterborne inputs to each of the basins. *BB* Bothnian Bay; *BS* Bothnian Sea; *GF* Gulf of Finland; *BP* Baltic Proper; *GR* Gulf of Riga; *DS* Danish straits; *KT* Kattegat. *Source* HELCOM (2013a)

Table 1 shows the reduction targets as specified in the 2007 and 2013 versions of the BSAP for each of the Baltic Sea’s seven sea basins: Bothnian Bay, Bothnian Sea, Gulf of Finland, Baltic Proper, Gulf of Riga, Danish straits and Kattegat (Fig. 1). Total reduction targets are generally lower in 2013 than in 2007 due to updated data and enhanced modelling tools. However, the Gulf of Finland has higher targets for both nutrients and the Baltic Proper for nitrogen in 2013 compared to 2007. Also worth noting, is that nitrogen reduction is no longer considered necessary in the Danish Straits. Overall, the 2013 BSAP targets put more emphasis on phosphorus than the 2007 BSAP targets.

**Table 1:** Annual reference nutrient loads and BSAP basin reduction targets in tonnes

Basin	Reference load 1997–2003		2007 BSAP reduction target		Reference load 1997–2003 (updated in 2013)		2013 BSAP reduction target	
	N	P	N	P	N	P	N	P
Bothnian Bay	51 440	2580	0	0	57 662	2675	0	0
Bothnian Sea	56 790	2460	0	0	79 372	2773	0	0
Gulf of Finland	112 680	6860	6000	2000	116 252	7509	14 452	3909
Baltic Proper	327 260	19 250	94 000	12 500	423 921	18 320	98 921	10 960
Gulf of Riga	78 400	2180	0	750	88 417	2328	0	308
Danish straits	45 890	1410	15 000	0	65 998	1601	0	0
Kattegat	64 260	1570	20 000	0	78 761	1687	4761	0
Total	736 720	36 310	135 000	15 250	910 344	36 894	118 134	15 177

*N* nitrogen, *P* phosphorus

*Source* HELCOM (2007b, 2013b)

The BSAP sets country-specific reduction targets for each basin (henceforth referred to as the country-and-basin targets) to achieve the overall basin targets specified in Table 1 (HELCOM 2007b, 2013a). The country-and-basin targets were set by using a two-step approach where they first calculated the load reduction that could be achieved by improved wastewater treatment for each country and basin, and then allocated the remaining load reduction among countries based on their proportion of the total load to each basin at the time.^1^ Hence, the country-and-basin targets were not designed to be cost effective. Instead, target setting was partly based on the polluter-pays principle.

Table 2 shows the total country targets set in both the 2007 and 2013 BSAP versions, i.e., the sum of the country-and-basin targets for each country. The updated 2013 BSAP brought large changes for the total country targets. For example, Denmark’s nitrogen reduction target was drastically reduced, due to the achievements made previously, while Finland’s target was doubled due to the higher nitrogen reduction required for the Gulf of Finland. Poland remains the country with the highest reduction target in the 2013 BSAP, but its target has been reduced compared to that of 2007.

**Table 2:** Country allocated reduction targets in tonnes of the Baltic Sea Action Plan

	2007 BSAP targets		2013 BSAP targets	
	Nitrogen	Phosphorus	Nitrogen	Phosphorus
Denmark	17 210	16	2890	38
Estonia	900	220	1800	320
Finland	1200	150	3030*	356*
Germany	5620	240	7670*	170*
Latvia	2560	300	1670	220
Lithuania	11 750	880	8970	1470
Poland	62 400	8760	43 610	7480
Russia	6970	2500	10 380*	3790*
Sweden	20 780	290	9240	530
Transboundary common pool	3780	1660		
Total	133 170	15 016	89 260	14 374

*Source* HELCOM (2007a, b, 2013b)

*Reduction requirements stemming from: 1. Finnish contribution to inputs from river Neva catchment, 2. German contribution to the river Odra inputs, based on ongoing modelling approaches with MONERIS, 3.Russian figures include contribution to inputs through Daugava, Nemunas and Pregolya rivers

A recent HELCOM report (HELCOM 2018b) describes the sources of nutrient inputs to the Baltic Sea. The main sources of nutrient inputs are direct point sources, atmospheric deposition, and rivers. Of the total nutrient inputs in 2014, 70% of nitrogen input and 95% of phosphorus input came from rivers. Natural background loads of nitrogen and phosphorus account for about one third of the total riverine loads. Diffuse sources, mainly from agriculture, account for 46% of the total riverine nitrogen load, and 36% of the phosphorus load. Point sources, mainly urban wastewater, account for 12% of the total riverine nitrogen load and 24% of the phosphorus load.

## Materials and methods

To synthesise the literature on how to cost-effectively reduce nutrient emissions to the Baltic Sea we use a systematic review methodology. What characterises a systematic review is that every stage is planned and documented in detail to maximise the transparency and reliability throughout the review process (Haddaway et al. 2015).

In order to find relevant literature that can answer our question we developed the review protocol by choosing search terms and literature databases. Identification of search terms is key to finding the relevant literature. Typically, systematic review is applied to fairly limited issues in the natural sciences. We were instead analysing a broad question and looking for papers in the social sciences, where a large number of studies studying the same issue normally does not occur. Hence, we did not want to be too restrictive when choosing our search terms. We needed terms that limited the regional scope of the search but allowed us to find papers focusing on any aspect of cost effectiveness of nutrient reduction to limit the risk of missing relevant studies. That is for a paper to be relevant it needed to be about (a) the Baltic Sea, (b) cost-effectiveness, and (c) the nutrients nitrogen or phosphorous; or eutrophication or reduction targets or measures which are other terms commonly used in this context. Our search terms were therefore: Baltic Sea, cost-effectiveness, cost-efficiency, nitrogen, phosphorus, nutrient, nitrate, eutrophication, reduction target, and measure. We subsequently combined the search terms as follows to define the search with logical restrictions:

"Baltic sea" AND (cost-efficiency OR cost-effectiveness) AND (nitrogen OR nutrient* OR nitrate* OR phosphorus OR eutrophication OR "reduction target*" OR measure*)

We searched three databases: Web of Science, Scopus and Google Scholar. When searching Google Scholar we used incognito mode to not affect the results by previous searches. The database search was carried out in September 2020 and it returned 47 results in Web of Science, 65 results in Scopus and 3160 results when using Google Scholar. We focused on the first 200 results from the Google search to make the task of screening the findings feasible. This strategy was considered acceptable as Google ranks search results on relevance. Many of the papers were found in more than one database. We also found 7 papers that were listed more than once in the same database. These duplicates were removed before the screening started.

In total, we had 232 search results to analyse. We reviewed titles, abstracts and publication type to make a selection of relevant studies. This was done by two researchers independently and the two researchers compared their results before relevant studies were chosen. For a paper to be selected in the first screening it needed to fulfil all of the following criteria:

i. The paper needed to be published in a book/peer-reviewed journal/working paper series or be a report published by an authority or organisation
ii. The paper needed to address cost effectiveness
iii. The paper needed to focus on the Baltic Sea region
iv. The paper needed to address eutrophication or nutrient emissions to the Baltic Sea
v. The paper needed not to be a working paper version of a published paper in the results list.

Among the search results from the three databases we found 102 papers that fulfilled all the criteria of the first screening. All of these papers were analysed in more detail to identify Baltic-wide studies that estimate the total costs of reducing nutrient emissions to the Baltic Sea. The second screening identified 32 relevant papers. The most common reasons why papers did not make the second screening were that they either evaluated the cost-effectiveness of a single nutrient reduction measure (typically construction of wetlands or mussel farming) or did not consider the entire Baltic Sea when evaluating different cost-effective nutrient abatement strategies.

Of the 32 selected papers, two were review papers (Elofsson 2010a; Halkos and Galani 2016), leaving 30 papers that provided new results on the total costs of nutrient reduction to the Baltics Sea from the database search. Next, we analysed references in the selected papers and found 3 additional studies that were relevant for our review. Consequently we identified 33 studies that investigate how to minimize the total costs of nutrient reductions to the Baltic Sea. All studies screened during the literature identification process can be found in Table S1 in the online supplementary material. To ensure that relevant literature was not missed we consulted with other participants in the BONUS TOOLS2SEA project, experts on cost-effective nutrient reductions to the Baltic Sea (see Acknowledgements) and attended BONUS-funded workshops that gathered both researchers and professionals from the Baltic Sea region, as well as presenting preliminary findings at the BONUS TOOLS2SEA workshop held back-to-back with the HELCOM AGRI group’s fall meeting in November 2019 in Berlin.

### The focus of the review

Generally, the 33 identified studies aim to find the mix of abatement measures that achieve a nutrient reduction target at the lowest total cost to society with the aid of a cost-minimization model. This is known as cost-effectiveness analysis (which in comparison to cost–benefit analysis does not require the valuation of environmental benefits, only the setting of environmental targets), the result of which is referred to as the cost-effective solution. In more technical terms, the cost-effective solution equalises marginal abatement costs of all abatement measures applied within the relevant catchment. If marginal costs are not equalised, there is potential for reaching the same target at lower cost by reallocating abatement among measures and/or locations (i.e., using more of those measures and/or locations with lower marginal abatement costs and less of those with higher costs).

The review of the selected papers focuses on potential general results in the literature that would be useful for future policy making. Hence, we do not aim to evaluate different cost-minimization models in detail. We are primarily interested in if it is possible to find general results concerning abatement measures and abatement strategies that are considered to be cost effective, and on how the abatement costs are allocated between countries.

Since the selected papers are published over a period of 20 years we expect to find differences between their results as models, data and examined reduction targets likely have changed over time. To obtain an overview of the literature, we first review the reduction targets that are examined and second, investigate whether any studies have used the same cost-minimization model. Mapping basic differences and similarities such as these between studies gives a better understanding of why results between studies may differ.

Third, we review which abatement measures that have been included in the analysis in the selected papers. We are also interested in the selected papers’ results on the cost-effectiveness of different measures. Hence, we investigate which abatement measures that have been selected to be part of the mix of measures recommended by the cost-minimization models’ low-cost solutions.

Fourth, we review the estimates of total costs for different reduction targets. We also review the different abatement strategies evaluated to determine if some are considered more cost effective than others. In particular we would like to know if some abatement strategies are seen as cost effective regardless of the reduction target examined, the choice of data and the model used.

Fifth and lastly, we review how the costs of abatement have been allocated between countries. That is, we focus on the costs of achieving the BSAP targets and expect to find that countries with relatively high reduction targets in Table 2 also bear a relatively high share of the cost burden. To round off the review, we investigate in “Cost allocation between countries” how the cost allocation between countries would be affected if the BSAP abatement strategy was replaced by more cost-effective abatement strategies, and in “Fairness in relation to the allocation of costs among countries” fairness in relation to the allocation of costs.

## Results of the primary literature review

In this section, we review differences and similarities between the selected studies and present general conclusions on how to reduce nutrient emissions in a cost-effective way.

### Reduction targets

Most of the reviewed studies either examine general percentage reductions, similar to the first reduction target agreed upon by HELCOM (see above), or different versions of the BSAP targets. Gren et al. (1997), Ollikainen and Honkatukia (2001) and Elofsson (2003) examine the cost of a 50% reduction of nitrogen and phosphorus emissions while Gren et al. (2008) investigate a range of reduction targets for both nitrogen (up to 50%) and phosphorus (up to 70%). A few studies focus on percentage reductions of nitrogen only. Elofsson (1999), Schou et al. (2006), Gren (2008a) and Czajkowski et al. (2019) examine nitrogen reductions varying between 20 and 50% in the different studies.

Eleven studies examine different ways to achieve the 2007 BSAP targets (Gren 2008b, c; Elofsson 2010b, c; Gren and Destouni 2012; BalticSTERN and SwAM 2013b; Gren et al. 2013; Ahlvik et al. 2014; Hyytiäinen et al. 2014, 2015; Wulff et al. 2014), while Hyytiäinen and Ahlvik (2015) and Hasler et al. (2014) focus on the 2013 BSAP targets. All these studies analyse both nitrogen and phosphorus reduction.

Some studies examine neither percentage targets nor the BSAP targets. Bryhn (2009) studies which phosphorus reductions are needed to restore the Secchi depths to their pre-1960s level. Hautakangas et al. (2014) examine the potential of wastewater treatment and to what extent wastewater treatment can fulfil the 2007 BSAP targets for both nitrogen and phosphorus. Häggmark Svensson and Elofsson (2019) perform an ex-post analysis of nitrogen reductions to the Baltic Sea and then use a cost-minimization model to show how the same reductions could have been achieved at a lower cost through reallocation of abatement measures among countries.

We also identify a group of papers that can be said to complement the above studies by analysing how nutrient-reduction cost estimates are affected when factors such as other environmental problems, learning-by-doing or novel measures are taken into account. Lindqvist et al. (2013), Gren and Säll (2015), Gren (2017), Nainggolan et al. (2018), and Gren and Ang (2019) study the costs of achieving water quality targets in the presence of climate change while Lindqvist and Gren (2013) and Elofsson (2014) instead focus on how technical change and knowledge diffusion affect total nutrient reduction costs. Gren et al. (2009) and Gren et al. (2018) examine how total costs are affected when an additional measure, mussel farming, is introduced. Both nitrogen and phosphorus are taken into account in the analysis in these studies. Note that the complementary studies are not the main focus of the review.

### Models used in most studies

All but two (Bryhn 2009; Hautakangas et al 2014) of the reviewed studies use some form of cost-minimization model that combines economic and ecological data (e.g. on retention, abatement costs and nutrient reduction effects) to estimate the total costs of nutrient reductions to the entire Baltic Sea. We find that many of the studies use the same model as the basis of the analysis. The models most often used are the Gren et al. (2008) model, the Gren et al. (2013) model, the BALTCOST model Hasler et al. (2012) and the MTT model (Ahlvik et al. 2014).

The Gren et al. (2008) model is used in five studies (Gren et al. 2008; Gren 2008b, c; Gren and Destouni 2012; Gren and Säll 2015) and the data on costs and effects of abatement measures provided in the paper have been used in other models as well (Elofsson 2010b, c; Gren et al. 2013). The model divides the Baltic Sea into 24 drainage basins for which emissions, costs and effects of abatement measures are calculated.

The BALTCOST model, building on the model developed by Schou et al. (2006), is used in five of the reviewed studies (BalticSTERN and SwAM 2013b; Hasler et al. 2014; Hyytiäinen et al. 2014; Wulff et al. 2014; Nainggolan et al. 2018). It employs 22 drainage basins for the optimization process. Relatively high-resolution spatial data, down to the 10 × 10 km^2^ grid level, is utilised for parameterising abatement cost and effect functions at the drainage basin scale (Hasler et al. 2014).

The MTT model developed by Ahlvik et al. (2014) is also used in five of the reviewed studies (BalticSTERN and SwAM 2013b; Ahlvik et al. 2014; Hyytiäinen et al. 2014, 2015; Hyytiäinen and Ahlvik 2015). The MTT model uses ecological marine modelling and accounts for feedbacks on load reductions caused by interdependencies between nutrients. It is a dynamic model which divides the Baltic Sea into 23 drainage basins. In comparison to a static model, the dynamic model can take the long-term effects of nutrient abatement into account. This can be especially important when analysing phosphorus reduction because the Baltic Sea responses are very slow with respect to changes in phosphorous loads (Boesch et al. 2006).

Lastly, the model developed in Gren et al. (2013) is the basis for the models in Lindqvist et al. (2013), Lindqvist and Gren (2013) and Gren (2017). Elofsson (2014) also draws on the Gren et al. (2013) model. The number of drainage basins used is 24 and the model takes nutrient transports between basins into account. As the MTT model, this model is dynamic.

### Abatement measures

We find that 22 different nutrient abatement measures have been included in the reviewed literature.^2^ These are applied in different sectors such as agriculture, energy and transport. No study includes all measures but most include measures from more than one sector. The exceptions are Elofsson (1999) and Czajkowski et al. (2019) that only include agricultural measures, and Hautakangas et al. (2014) that focus on wastewater treatment. Table 3 shows how many studies that have used a certain measure and if the measure has been applied to reduce nitrogen, phosphorus or both. We find that some measures are more common than others. Improved urban wastewater treatment, reductions in livestock, cultivation of catch crops, reduction in fertilization, and restoration/construction of wetlands are used in almost every study. The general focus on agricultural measures and wastewater treatment is expected since these sectors are the main emitters of nutrients to the Baltic Sea.

**Table 3:** List of measures

Measure	Number of studies	N	P	Measure	Number of studies	N	P
Catalysts in cars	5	x		Buffer strips	13		x
Catalysts in ships	16	x		Change in the spreading time of manure	18	x	
Catalysts in trucks	11	x		Cultivation of catch crops	29	x	x
Catalysts in power plants	16	x		Energy forestry	20	x	x
Construction of sedimentation ponds	5		x	Fallow with cover crop	2	x	
Mussel farming	4	x	x	Fertiliser reduction	30	x	x
Wetlands	28	x	x	Grasslands	20	x	x
Improved urban wastewater treatment	30	x	x	Reduction of cattle	31	x	x
P-free detergents	17		x	Reduction of pigs	31	x	x
Private sewers	14	x	x	Reduction of poultry	25	x	x
Soil drainage	1		x	Winter crops	2	x	

Note that most measures abate both nutrients and that studies using the same model include the same abatement measures in their cost estimations. For example, studies using the BALTCOST model include the same six measures (wastewater treatment, wetlands, catch crops, N fertilizer reduction, reduction of cattle and reduction of pigs), and studies using the MTT model include the same 9 measures (wastewater treatment, P-free detergents, sedimentation ponds, wetlands, catch crops, N & P fertilizer reduction, reduction of cattle, reduction of pigs and reduction of poultry), as can be seen in Table S2. An important point is also that the most recent studies focusing on the BSAP targets tend to include fewer abatement measures than the older studies focusing on percentage targets. It could be counterintuitive that newer models include fewer measures than older models as data access tend to improve over time. However, the newer models tend to be more computationally advanced and demand data of higher precision.

Generally speaking, a cost-effective nutrient abatement measure is a measure that gives a lot of reduction per euro spent, i.e. the cost per tonne of nitrogen or phosphorus reduced from the sea is relatively low. The cost-minimization models in the reviewed studies choose the mix of abatement measures that minimize the total costs of reaching a specific reduction target. For low reduction levels, it may suffice to only use the cheapest measures but for more ambitious targets, the capacity constraints or diminishing returns of the cheaper measures may imply that also more expensive measures are needed in the cost-effective solution suggested by the model. We have examined which measures that tend to be part of the studies’ cost-effective solutions. These measures are, hence, cost-effective in relation to the target the model is trying to achieve. We here focus on measures that have been recommended for achieving separate 50% reductions of nitrogen and phosphorus and for achieving the BSAP targets.

Gren et al. (1997, 2008) and Elofsson (1999, 2003) report which measures that are used for achieving a 50% nitrogen reduction. All studies recommend reduction of nitrogen fertilization and all, but Elofsson (1999) who focus on agricultural measures, also recommend improved urban wastewater treatment as a cost-effective measure. Cultivation of catch crops is part of the cost-effective solution in three cases (Gren et al. 1997, 2008; Elofsson 1999) but Gren et al. (2008) show that it is mainly used when other more cost-effective measures have reached their capacity. Other measures included in the cost-effective solutions are wetlands (Gren et al. 1997, 2008), change in the spreading time of manure (Elofsson 1999; Gren et al. 2008), cultivation of winter crops (Elofsson 1999) and cultivation of ley grass (Elofsson 1999). Measures to reduce air emissions generally play a minor role but can contribute to the cost-effective solution (Gren et al. 1997, 2008).

Gren et al. (1997, 2008) and Elofsson (2003) report which measures that are used for achieving a 50% reduction of phosphorus. They all include improved urban wastewater treatment and reduction of phosphorus fertilization in their cost-effective solutions. Gren et al. (1997, 2008) also highlight wetlands as a potentially cost-effective measure for phosphorus reduction. Additionally, Gren et al. (2008) include P-free detergents and cultivation of catch crops as measures of minor importance in the cost-effective solution. Elofsson (2003) only suggests land use changes if the phosphorus target is to be met with high certainty. Note that quite a few of the included measures in Gren et al. (1997, 2008) and Elofsson (1999, 2003) are never selected for the cost-effective solution, neither when nitrogen or phosphorus reductions are examined. Examples of such measures are reductions of livestock, buffer strips and energy forestry.

Studies reporting recommended measures for achieving the 2007 BSAP targets use either the BALTCOST (Hyytiäinen et al. 2014; Wulff et al. 2014) or the MTT model (BalticSTERN and SwAM 2013b; Ahlvik et al. 2014; Hyytiäinen et al. 2015). Both models’ suggested solutions include all measures available to achieve the target set (BalticSTERN and SwAM 2013b; Wulff et al. 2014) but some measures are used to a larger extent.^3^ Improved urban wastewater treatment is the most important measure for phosphorus abatement and it also contributes to a large share of the needed nitrogen reduction regardless of which model is used (Ahlvik et al. 2014; Hyytiäinen et al. 2014, 2015). Reduction of nitrogen fertilization and restoration of wetlands are important for cost-effective nitrogen abatement in both models’ solutions (BalticSTERN and SwAM 2013b; Hyytiäinen et al. 2014). Using the MTT model, BalticSTERN and SwAM (2013b) shows that reduction of phosphorus fertilization, P-free detergents and phosphorus ponds are also relatively cost-effective phosphorus abatement measures but have limited capacity. Reductions of livestock are included in both models’ solutions, due to the lack of alternative measures, but are found to be very expensive (Hyytiäinen et al. 2014).

Results are similar when the 2013 BSAP targets are examined in Hasler et al. (2014) and Hyytiäinen and Ahlvik (2015). Improved urban wastewater treatment is the most important measure for achieving the target cost effectively both when using the MTT model (Hyytiäinen and Ahlvik 2015) and the BALTCOST model (Hasler et al. 2014), while restoration of wetlands (Hasler et al. 2014) and other retention measures such as phosphorus ponds (Hyytiäinen and Ahlvik 2015) also are deemed as relatively cost effective. The BALTCOST model is not able to achieve the phosphorus target in all basins despite using all phosphorus measures available in the model (Hasler et al. 2014). Since the phosphorus target is difficult to reach, the model prioritises measures that abate phosphorus (wastewater treatment, wetlands, and reductions in livestock) and deliver simultaneously enough nitrogen abatement to reach the nitrogen target. Potentially cost-effective nitrogen measures (e.g. reduction of nitrogen fertilization) that have no or limited impact on phosphorus emissions are, therefore, rarely chosen by the model. Note that this effect is stronger when examining the 2013 BSAP targets, since these put more emphasis on phosphorus abatement than the 2007 BSAP targets, as seen in Table 1. The importance of phosphorus abatement and the fact that many phosphorus measures also reduce nitrogen concentrations in the Baltic Sea are also highlighted in Gren et al. (2013), Ahlvik et al. (2014), and Hyytiäinen et al. (2015) when examining the 2007 BSAP targets. For example, Gren et al. (2013) find that reaching the phosphorus target for the Baltic Proper is the most important target as this would simultaneously achieve the nutrient pool target in almost all other basins.

This suggests that investing in relatively cheap abatement measures focusing on phosphorus reduction that additionally deliver nitrogen abatement, could be a prudent step forward especially in basins with a high phosphorus load such as the Baltic Proper. According to the review of recommended measures, improved wastewater treatment is the most important measure for cost-effective phosphorus abatement. Hyytiäinen et al. (2015) find that that the optimal level of water quality protection can be reached mainly by investing in wastewater treatment in the sub-catchments draining to the Baltic Proper, the Gulf of Riga and the Gulf of Finland. Similarly, Hautakangas et al. (2014) show that improved wastewater treatment alone can result in 70% of the BSAP nitrogen target and 80% of the phosphorus target.

As shown above, improved wastewater treatment will need to be combined with other measures that are cost effective and have relatively high capacity if the nutrient reduction targets are to be achieved. Our review shows that wetlands and reduction of phosphorous fertilization^4^ are often selected to contribute to cost-effective phosphorous abatement. Phosphorus ponds is another phosphorus abatement measure that shows potential. Reduction of nitrogen fertilization and wetlands are often selected to contribute to cost-effective nitrogen abatement. That expensive measures such as reductions in livestock are sometimes used to achieve the BSAP targets highlights the need to include additional measures, especially those that focus on phosphorus, in the models. The effect of changing the spreading time of manure on phosphorous abatement has, for example, been neglected, as shown in Table 3.

### Total costs of nutrient abatement and cost-effective abatement strategies

Naturally, cost estimates differ between studies due to the differences in targets, models and data used. Comparisons of cost estimates are therefore difficult, but highly desirable to provide an idea of the size and structure of costs in relation to alternative abatement strategies, which is a main aim of this literature. Detailed information on all selected studies and their cost estimates can be found in Table S3 in the online supplementary material. To save space, we here focus on the costs of achieving the 2007 and 2013 BSAP targets since these are the most recent targets set by HELCOM and the most relevant for future policy making.

The estimates show that achieving the 2007 BSAP targets costs between 1.4 and 4.7 billion EUR annually, (Gren 2008c; Gren et al. 2009, 2013; Elofsson 2010c; Gren and Destouni 2012; BalticSTERN and SwAM 2013b; Ahlvik et al. 2014; Wulff et al. 2014; Hyytiäinen et al. 2015) while achieving the 2013 BSAP targets costs between 1.5 and 4.2 billion EUR annually (Hasler et al. 2014; Hyytiäinen and Ahlvik 2015; Gren et al. 2018; Nainggolan et al. 2018; Gren and Ang 2019). Hence, there are large differences in cost estimates between studies even when the same target is examined. However, a large part of the cost differences can be explained either by the choice of baseline loads, as the baseline defines how much reduction is needed to reach the target, or the choice of abatement strategy.

Marginal costs rise rapidly with increases in the reduction ambition (Gren 2008b; Czajkowski et al. 2019). Even small changes in the baseline loads, due to past reduction efforts, can therefore give profound effects on costs. This is confirmed in Gren and Destouni (2012) who specifically examine the effect on total costs of using different baseline loads. Hyytiäinen et al. (2014) also experiment with different loads and find that achieving the 2007 BSAP basin targets using the BALTCOST model and years 1997–2003 as the baseline for initial nutrient loads costs 4.7 billion EUR annually. Achieving the same targets using the same model but years 2004–2008 as the baseline for initial loads costs only 1.4 billion EUR annually.

Evidently, using recent data, for which the initial nutrient load is lower due to past nutrient reduction effort, significantly reduces costs as less reduction is needed. On the other hand, one could expect that using recent data would lead to higher cost estimates if past reduction has focused on the cheapest abatement measures. We only find one study among the reviewed literature claiming this to be an issue (Gren and Säll 2015) and we do not find evidence for general increases in total abatement cost estimates over time. Moreover, Häggmark Svensson and Elofsson (2019) show that net reductions in nitrogen emissions in the Baltic Sea region achieved through environmental policy between the periods 1992–1996 and 2008–2010 could have been obtained at only 12% of the realised cost, if abatement had been reallocated cost-effectively among countries. This suggests that nutrient reduction in the past has been far from cost effective.

Another reason why cost estimates differ that is not dependent on the choice of data or model, is that different abatement strategies have been examined. When reviewing different abatement strategies and their costs we have identified important factors for cost-effective nutrient abatement. First, simultaneous reduction of nitrogen and phosphorus is considerably cheaper than independent reductions since many abatement measures target both nitrogen and phosphorous. For example, Gren et al. (2013) show that the total saving of simultaneous reduction for achieving the 2013 BSAP targets is 25 billion EUR over a period of 70 years. The benefits of simultaneous reduction is also demonstrated in studies focusing on percentage targets (Gren et al. 1997, 2013; Gren 2000; Elofsson 2003). Further, it is more cost-effective to consider several environmental problems simultaneously than separately. Considering nutrient and greenhouse gas emissions simultaneously is therefore recommended (Gren and Säll 2015; Nainggolan et al. 2018).

Second, it has been suggested that nutrients should be reduced in all Baltic Sea basins to be able to achieve the BSAP targets in a more cost-effective way. Estimations in Ahlvik et al. (2014) and Gren (2008c) show that nutrient reductions in the Bothnian Bay and Bothnian Sea are part of the cost-effective solution, even if it is not required in the BSAP, as those have positive effects on water quality in the Baltic Proper, the basin with the largest nutrient reduction need.

Third, and most importantly, cost-effective abatement depends on the spatial scale at which the targets are set, i.e. to what extent countries can cooperate to achieve the targets. Already the very first Baltic-wide studies, Gren et al. (1997) and Ollikainen and Honkatukia (2001), showed that proportional reductions, i.e. that all countries reduce emissions by a similar percentage, is inefficient compared to overall reductions of the same magnitude. Similar conclusions are found in studies analysing the BSAP targets. Gren (2008b, c), Elofsson (2010c), BalticSTERN and SwAM (2013b), Hyytiäinen et al. (2014), and Hyytiäinen et al. (2015), Gren et al. (2018) and Hyytiäinen and Ahlvik (2015) find that substantial cost savings can be made if the BSAP targets were designed at a coarser spatial scale than the current country-and-basin scale (e.g. basin scale, or sea scale).

The abatement costs of different measures vary spatially and this could be taken advantage of when designing the abatement strategy for a particular basin. Setting targets for each country to each basin, as the BSAP does, is excessively expensive, because it makes it necessary to use costly abatement measures that are not particularly effective. If abatement strategies were more flexible, e.g. set at the basin scale, countries could cooperate and use the measures that give the most abatement for the least cost in each basin regardless of country borders. For example, Hyytiäinen and Ahlvik (2015) show that 500 million EUR could be saved annually if the 2013 BSAP targets were set on a basin scale, instead of country-and-basin, and allowed countries to be credited for reductions obtained in adjoining sea basins. Czajkowski et al. (2019) also experiment with the spatial scale at which the targets are set, ranging from the entire Baltic Sea down to grid square level. Although their focus is nitrogen reduction only, a coarser spatial scale for targets and allowing for cooperation between countries, is found to be associated with lower costs.

We conclude that the current BSAP, with specific country-and-basin targets, must be regarded as cost inefficient, as the same overall reduction can be achieved at a far lower cost. However, introducing cost-effective BSAP targets would mean a change in the spatial allocation of nutrient abatement measures, which could affect the relative cost burdens among countries. The division of costs between countries and how it would be affected by more cost-effective abatement strategies is reviewed next.

## Cost allocation between countries

Most of the reviewed studies have analysed the division of the costs of nutrient reduction between countries. This means that they offer cost estimates per country of achieving a particular reduction target. In this section, we review how the cost burden of reducing nutrient emissions based on 2007 and 2013 BSAP targets or more cost-efficient solutions has been allocated among countries.

Ten of the reviewed studies have reported the allocation of costs of achieving the 2007 BSAP targets among countries (Gren 2008b, c; Elofsson 2010b, c; Gren and Destouni 2012; BalticSTERN and SwAM 2013b; Gren et al. 2013; Hyytiäinen et al. 2014, 2015; Wulff et al. 2014). Poland carries the highest cost burden in all studies. This is not surprising since Poland also has the largest reduction target, as shown in Table 2. However, Poland’s share of the total costs varies considerably from 27% (Hyytiäinen et al. 2015) to 80% (Gren 2008b). Denmark, Germany, Russia and Lithuania are reported as the next largest payers but the results vary between studies. In all studies, the costs are found to be lowest for Finland and Estonia.

**Fig. 2:**
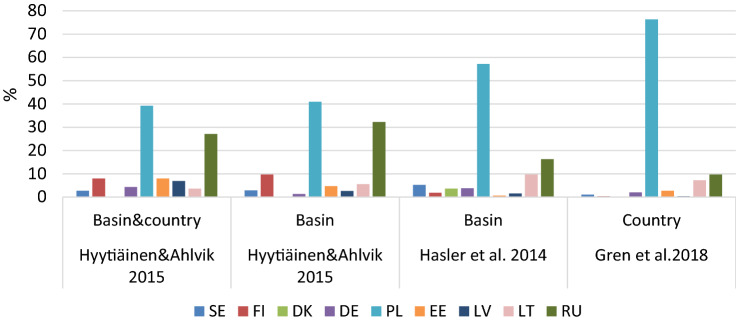
Allocation of costs of nutrient reduction among littoral countries, represented as percentages of the total costs. Cost shares are represented for basin and/or country targets according to 2013 BSAP based on Hyytiäinen and Ahlvik (2015), Hasler et al. (2014) and Gren et al. (2018). Country codes are as follows: SE (Sweden), FI (Finland), DK (Denmark), DE (Germany), PL (Poland), EE (Estonia), LV (Latvia), LT (Lithuania), RU (Russia)

The cost burden of the 2013 BSAP targets are analysed in Hyytiäinen and Ahlvik (2015) for country-and-basin targets as well as basin targets, in Hasler et al. (2014)^5^ for basin targets, and in Gren et al. (2018) for country targets. Differences between these studies’ results can be expected since Hasler et al. (2014) and Gren et al. (2018) use static models while Hyytiäinen and Ahlvik (2015) use the dynamic MTT model. In addition, different initial loads and thus amounts of reductions have been used, see Table S3 in the online supplementary material. In Fig. 2, we show results on cost allocations. According to Hasler et al. (2014), Poland’s cost share is 57% but Hyytiäinen and Ahlvik (2015) estimate it to be about 40%. The highest cost share of nearly 80% is found in Gren et al. (2018). Russia’s reduction target was substantially increased with the update of the BSAP in 2013, see Table 2. As a result, Russia’s share of the total cost of abatement has increased (see e.g. Hyytiäinen et al. 2015). The cost burden of Denmark, on the other hand, is close to zero as the reduction targets for the Danish Straits and Kattegat basins have been drastically reduced compared to the 2007 targets.

**Fig. 3:**
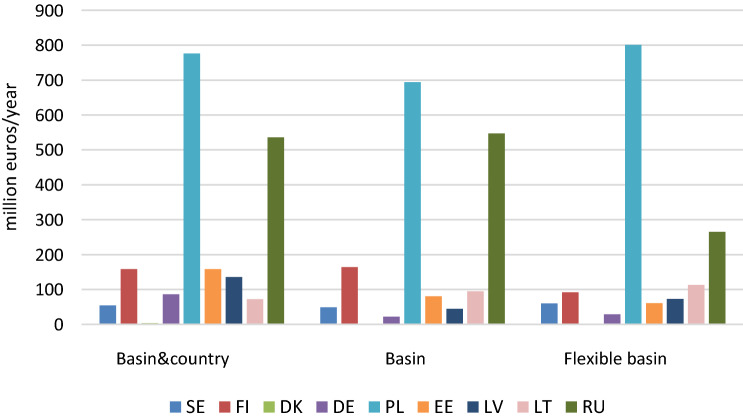
Abatement expenditures per country with different flexibilities: basin-and-country targets, basin targets and basin targets with nutrient exchange, million euros annually. Country codes are as follows: SE (Sweden), FI (Finland), DK (Denmark), DE (Germany), PL (Poland), EE (Estonia), LV (Latvia), LT (Lithuania), RU (Russia). *Source* Hyytiäinen and Ahlvik (2015)

To compare the cost allocation of achieving the BSAP targets with more cost-effective solutions, we consider the studies of Hyytiäinen et al. (2015), Hyytiäinen and Ahlvik (2015), Elofsson (2010b), and Gren et al. (2018). As an example, the cost allocations for country-and-basin targets, basin targets and flexible basin targets from Hyytiäinen and Ahlvik (2015) are presented in Fig. 3. A country may incur higher costs in the cost-effective solution compared to BSAP if it has low-cost measures available that are not being utilized with the BSAP solution. Indeed, higher costs are obtained for 2–4 countries when moving from country-and-basin-targets to more cost-effective solutions. The figure illustrates that costs may first increase when moving from country-and-basin targets to basin targets and then decrease when moving to the most cost-efficient solution with flexible basin targets. That is the case for Russia, while opposite impacts are found for Poland. In Hyytiäinen and Ahlvik (2015) most of the cost savings (500 million EUR annually) from moving from 2013 BSAP targets to basin targets with nutrient exchange occur from reallocation of abatement from the Gulf of Finland to the Baltic Proper; and therefore increasing the cost burden for Lithuania and Poland. Finland, Estonia, Germany, Sweden and Latvia may also face higher cost burdens in the cost-effective solution but results differ between studies. The absolute increases are rather small but in relative terms more significant due to the reduction in total costs. It should be noted that the abatement undertaken in a given country would not necessarily be paid for by that country; with joint implementation there would be opportunities for financial transfers.

## Fairness in relation to the allocation of costs among countries

Perceptions of the fairness of the allocation of costs for improving the state of the Baltic Sea are essential for legitimizing decision making and hence motivating countries to comply with their BSAP commitments (Birnbaum et al. 2015). In the following we review three approaches to assessing fairness and countries’ willingness to participate in the BSAP: (i) criteria that relate abatement costs to population or GDP, (ii) the cost–benefit approach and (iii) the game-theoretical approach that deals with countries’ strategic behaviour and the need for side-payments in order to reach agreed targets.

Fairness criteria have philosophical grounds in equal human rights or abilities to bear the financial burden of nutrient abatement. A large number of distributive fairness or equity principles have been suggested in the literature. Commonly referred to and applied principles include the egalitarian principle, the sovereignty principle and the ability-to pay principle (Rose et al. 1998; Ringius et al. 2002; Lange et al. 2007; van den Berg et al. 2019). The egalitarian principle is based on the idea of the equal worth of all humans. Thus, it implies that each individual has an equal right to pollute and allocates emissions allowances relative to the population. The sovereignty principle is also based on rights to pollute, but relates them to the nations’ historical levels of emissions (e.g. grandfathering of emission rights).

The ability-to-pay, or capability, principle is based on the ability of countries to bear the economic burden of abatement. The most commonly used metric for ability-to-pay is to relate the economic burden to per capita GDP. These principles can be further developed, for example the egalitarian principle may also take equity between generations into account. The distributive fairness criteria stand in stark contrast with the polluter pays principle that does not consider the relative economic burden, but only the absolute levels of emissions (Ringius et al. 2002; Lange et al. 2007), as implied by the BSAP.

In the climate policy literature, equity principles have been widely studied. Ringius et al. (2002) find that different nations emphasize different equity principles and suggest that more complex formulae that consider aspects of all principles are needed. According to the survey and econometric analysis performed in Lange et al. (2007), the polluter pays principle was the most universally accepted equity principle among countries, though less favoured by rich countries. Further, over the long run, attitudes towards egalitarian principles tend to strengthen and economic self-interest to weaken. Recently, the literature has focused on how country level emission targets and carbon budgets can be derived based on equity criteria (see e.g. van den Berg et al. 2019).

In the case of the Baltic Sea literature, the fairness criteria approach has only been applied in Gren (2008b) and in Gren and Destouni (2012) for the 2007 BSAP targets. Gren (2008b) examines loads per capita as an example of the egalitarian principle, and costs related to GDP per capita as an example of the ability-to-pay principle, finding considerable variation among countries in all scenarios examined. According to Gren (2008b), the loads per capita vary from 6–40 kg for nitrogen and 0.1–0.9 kg for phosphorus according to the BSAP while the variation is 6–31 kg N and 0.1–0.6 kg P in the cost effective solution. The abatement costs are 0–1.9% of GDP in the drainage basin area in the BSAP scenario and 0–1.3% of GDP in the cost effective solution. While the costs are largest for Poland in absolute terms, the relative costs are highest for Lithuania due to its low GDP. The group of countries for which the agreement could be perceived as unfair is therefore different for different fairness criteria. In Gren (2008b) the main focus is the fairness of the BSAP relative to other abatement scenarios. It is found that according to the equal-loads-per-capita criterion, the BSAP does not improve fairness compared to the case without nutrient reductions or the cost-effective solution. On the other hand, according to the equal-abatement-costs-per-capita and ability-to-pay criteria the BSAP is fairer than the cost-effective solution. Gren and Destouni (2012) adapt the same equity principles as in Gren (2008b) while expanding the analysis by using the Gini-index to measure inequality. They demonstrate that relatively poor countries face the largest economic burdens, and that this inequitable distribution of costs is independent of the investigated differences in nutrient load measurements. Hyytiäinen et al. (2015) also present costs as percentages of GDP for the 2007 BSAP targets. They find that the costs per unit GDP are highest for Latvia, Lithuania, Denmark, Estonia and Poland. Unfortunately, no study on fairness of the 2013 BSAP targets exists.

Cost–benefit analysis has been applied in Hyytiäinen et al. (2015) to weigh the costs of different nutrient abatement strategies against the benefits of improved water quality. The benefits are based on willingness-to-pay studies in all littoral countries (but are likely to be underestimated as they exclude the benefits related to healthier inland waters). The results show that benefits are unevenly distributed among countries, but that overall benefits exceed the costs under all strategies, including the one corresponding to the 2007 BSAP targets. Results indicate that the 2007 BSAP is favourable for Finland, Sweden, Germany and Russia as their benefits exceed their costs. Conversely, for Poland, Denmark and the Baltics their costs exceed their benefits. The grouping of countries into winners and losers remains unchanged when shifting from country-and-basin targets towards basin targets or flexible targets. The study also suggests that in order to make an agreement attractive for all countries, it is better to maximize overall net benefits from improved water quality and have the winners compensate the losers using side-payments, rather than adjusting the abatement targets so that the benefits would outweigh the costs for each country.

Although comparing costs and benefits provides some indication of the willingness of countries to join an agreement, game-theoretic analyses can help to better understand the incentives faced by the different countries to commit to an agreement while taking into account whether the other countries are likely to do their share. Game-theoretic analyses, such as Ahlvik and Pavlova (2013), utilize information on the costs and benefits of reduced nutrient loads to analyse the strategic behaviour of littoral countries and the need for side-payments to reach a stable agreement. They first analyse an agreement in which nutrient loads are reduced to the socially optimal level. They find that for most of the countries, the costs exceed the benefits, implying that they have an incentive to deviate from an agreement. Countries with higher benefits than costs have, on the other hand, an incentive to offer side payments to other countries to support their participation. However, according to the model results, their surplus was not large enough to compensate the losers so cooperation could not be supported. Second, they find that a treaty with all countries involved and modest abatement targets is preferable to a treaty with more ambitious targets for fewer countries, in terms of higher net benefits and lower total nutrient load. Thus, their study does not support the view that multiple coalitions with few motivated countries might achieve more than a single coalition, presented in Roggero et al. (2019) based on the literature on international environmental agreements. The stability of the international agreement regarding eutrophication in the Baltic Sea has also been studied in Markowska and Zylicz (1999). They find that full co-operation between littoral countries in the case of a joint 50% reduction in the nitrogen load can be stabilized with a side payments scheme in which Germany, Poland and the Baltics are net receivers. Consequently, the studies highlight the importance of side payments for obtaining the analysed agreements.

Our literature review shows that neither BSAP nor cost-effective allocations of nutrient load abatement are fair, and hence there is likely to be a need for side payments. The questions remain which countries should receive side payments, what is the fair amount of side payments and what is the most suitable payment mechanism. The grouping of countries in winners and losers differs between the approaches and fairness criteria. According to the ability-to-pay criteria, side payments should be paid to countries with high abatement cost and relatively low GDP. On the other hand, the cost–benefit approach suggests providing compensation to countries where their costs exceed their benefits. However, the valuations of benefits are generally characterised by relatively high uncertainty compared to quantification of costs.

## Concluding discussion

This paper has reviewed Baltic-wide studies focusing on cost-effective nutrient reductions to the Baltic Sea. Although cost estimates of achieving the BSAP targets differ between studies, and are not completely comparable, it is clear that considerable costs are associated with reducing nutrient emissions. Unfortunately cost effectiveness was not taken into consideration when designing the BSAP targets. When targets are designed as in the BSAP, where countries are assigned specific targets for different basins, avoiding expensive ineffective abatement measures may be impossible. If countries would cooperate and find the least-costly abatement strategy for a certain basin or the entire sea, costs would be reduced according to all the studies reviewed here. Hence, the current BSAP is not cost effective and our review is conclusive in that the same overall reduction could be achieved at considerably lower cost if more flexible abatement strategies were used.

If cost-effective nutrient abatement is to be achieved, cost-effective abatement measures should be prioritised. According to the model simulations investigated in this paper such measures are improved wastewater treatment, construction of wetlands, and reduction of nitrogen and phosphorus fertilisation. However, the cost-minimisation models used in the studies recommending measures for cost-effective achievement of the BSAP only include a few of the abatement measures possible to apply in the Baltic Sea region. Identifying cost-effective measures not presently included in the latest model studies, in particular phosphorous measures, is therefore an urgent task for future research to increase the precision of recommendations. Hasler et al. (2012) and Wulff et al. (2014) suggest, for example, to introduce improved manure handling and buffer zones into the BALTCOST model if data would become available. Considering the implementation shortfalls in manure storage capacity in several countries identified by Thorsøe et al. (2021) and that adequate manure storage and application techniques are critical prerequisites for efficient use of manure in crop production (Svanbäck et al. 2019), it is unfortunate that the effect that improved timing in manure spreading might have on phosphorous abatement has been understudied (see Table 3). More research is needed to see if this is a cost-effective way to abate phosphorous. Manure management measures such as investments in greater manure storage capacity and precision spreading technology are also highlighted as effective nitrogen abatement measures by Jansson et al. (2019). Gypsum treatment is another potentially interesting measure for phosphorus reduction (Ekholm et al. 2012; Uusitala et al. 2012) not yet included in any of the reviewed models.

Naturally, the number of abatement measures included in the cost-minimisation models affects outcomes and cost estimates. If only a few measures are included, the total cost of abatement is likely to be higher as there is a greater risk that expensive measures must be used to achieve the target, as seen in Hasler et al. (2014) and Hyytiäinen et al. (2015). Consequently, it is likely that the estimated least cost(s) of abatement to restore the Baltic Sea to good health can be reduced even further if the omitted abatement measures were included in the simulation models. To facilitate the inclusion of additional measures in the models, more resources should be devoted to data collection on the costs and effectiveness of abatement measures in different locations.

What is considered a cost-effective measure differs between locations. The cost-effectiveness models take spatial variability into account but all models are designed at a large scale. Typically, the models divide the Baltic Sea region into 20–25 catchments (e.g. 24 catchments in Gren et al. 2008; 23 catchments in Ahlvik et al. 2014). The Czajkowski et al. (2019) model is the model with the highest resolution, still their 10 × 10 km^2^ grid resolution is relatively large given the characteristics of nutrient emissions from agricultural land. The large scale means that the models mainly provide a general overview of which measures that could be suitable in different areas. For instance, it is clear that improved wastewater treatment is mainly cost-effective in areas where investments have not already been made such as the Eastern Baltic region (e.g. Hyytiäinen et al. 2015). It is well known that the cost effectiveness of agricultural measures is highly context dependent (Jacobsen and Hansen 2016; Shortle and Horan 2016). This means that there could be large variations in the cost effectiveness of measures within a catchment, variations that are not captured with the models reviewed in this paper. Hence, another type of analysis is needed to find which measures are cost-effective for a particular farm. Analyses at the farm or even field level are, therefore, necessary to complement the Baltic-wide studies in order to guide selection of the best nutrient abatement measures.

When interpreting the results of the model simulations it is important to keep in mind that the cost estimates can never provide an exact answer to what abatement will actually cost, it is a qualified assessment based on the knowledge and data that are presently available. Further, the cost-minimisation models do not explicitly take benefits of reduced nutrient emissions into account. Reduced nutrient emissions will have positive effects on the Baltic Sea ecosystem but will also provide additional benefits such as reduction of greenhouse gases (Gren and Säll 2015; Nainggolan et al. 2018; Gren and Ang 2019), improved ground water quality (Wezel et al. 2016) and reduced exposure to toxic metals (Pizzol et al. 2014). All these benefits may be difficult to value, but estimates can be derived from the published literature. Since restoring the water quality of the sea takes time there is an additional difficulty in valuing the benefits of restoring the ecosystem because people tend to value the present higher than the future. As shown in Hyytiäinen et al. (2015), countries also differ in their citizens’ valuations of a clean Baltic Sea. Whether or not reducing nutrient emissions is worth the cost is therefore ultimately a political question. Nevertheless, cost-effective reduction of nutrient emissions would increase the chances of reaching a good environmental status of the sea.

Fairness needs to be considered to increase the willingness of countries to meet their commitments to reducing eutrophication in practice. Moving towards the cost-effective solution will change the spatial distribution of abatement and increase efforts in some countries with relatively low GDP. The review of model results showed that neither the BSAP allocation nor the cost-effective solution is fair and that side-payments are necessary to achieve a fair distribution of abatement costs among countries. Thus, a mechanism for winners compensating losers needs to be established to improve the political feasibility of achieving HELCOM goals (cf. Brady et al. 2021).

In conclusion, the potential for restoring the Baltic Sea to good health is currently undermined by an abatement strategy, BSAP 2013, that is more costly than necessary and an allocation of the abatement cost burden among the littoral countries that is likely to be perceived as unfair by several countries. It is conclusive from this systematic review and synthesis of the literature that nutrient abatement can be achieved at lower cost than the current strategy, BSAP 2013. The main question now, given the insufficient progress in reducing nutrient emissions, is how to get countries and individual farmers to change their behaviour and practices so that the Baltic Sea can be restored. In order to improve the likelihood of full commitment to HELCOM goals, and hence success, the future BSAP needs to consider both cost-effectiveness and fairness as well as additional low-cost abatement measures.

## Supplementary Information

Below is the link to the electronic supplementary material.

Supplementary file1 (PDF 567 kb)

## Footnotes

1 For information on exact calculations, the interested reader is referred to HELCOM (2007b, 2013a).

2 A list of all studies and their measures can be found in Table S2 in the online supplementary material.

3 The BALTCOST model still does not achieve the phosphorus target in all basins but the nitrogen reduction target is exceeded (Wulff et al., 2014).

4 According to Hyytiäinen et al., (2014), reduction of phosphorus fertilization is especially recommended in Finland, Germany and Denmark.

5 In Nainggolan et al. (2018), the cost estimates without climate change are the same as reported in Hasler et al. (2014).
