# Supplementary material for: How unnecessarily high abatement costs and unresolved distributional issues undermine nutrient reductions to the Baltic Sea
Source: Ambio. 2021 Jun 9;51(1):51–68. doi: 10.1007/s13280-021-01580-4 (PMC8651968; doi:10.1007/s13280-021-01580-4)
Supplement: Supplementary file 1 — Supplementary file1 (PDF 567 kb) [file 13280_2021_1580_MOESM1_ESM.pdf]

***Ambio***

Electronic Supplementary Material
*This supplementary material has not been peer reviewed.*

Title: **How unnecessarily high abatement costs and unresolved distributional issues undermine nutrient reductions to the Baltic Sea**

Table S1 Search results

A = 1 paper was selected in the first screening. B = 1 paper was selected in the second screening.

| A | B | Search results | Web of Science | Scopus | Google scholar |
|---|---|---|---|---|---|
| 1 | 0 | Ackermann, A., Mahnkopf, J., Heidecke, C., & Venohr, M. (2016) Reducing agricultural nitrogen inputs in the German Baltic Sea catchment – trends and policy options, Water Science and Technology 74(5): 1060-1068. | | | yes |
| 1 | 1 | Ahlvik, L., Ekholm, P., Hyytiäinen, K., & Pitkänen, H. (2014) An economic–ecological model to evaluate impacts of nutrient abatement in the Baltic Sea, Environmental Modelling & Software 55: 164-175. | yes | yes | yes |
| 0 | | Ahlvik, L. (2010) Cost-effective nutrient abatement in the Baltic Sea, Master's thesis. | | | yes |
| 0 | | Ahlvik, L., Hyytiäinen, K., Rankinen, K. LIFE12 ENV/FI/592. | | | yes |
| 1 | 0 | Akram, U., Quttineh, N.H., Wennergren, U., Tonderski, K., Metson, G.S. (2019) Optimizing nutrient recycling from excreta in Sweden and Pakistan: Higher spatial resolution makes transportation more attractive, Front. Sustain. Food Syst. 3(50). | | | yes |
| 0 | | Alam, K., Rolfe, J., & Donaghy, P. (2008) Assessing the cost-effectiveness of water quality interventions in South-East Queensland, Australasian Journal of Environmental Management 15(1): 30-40. | | | yes |
| 0 | | Andreoni, V., Miola, A., Perujo, A. (2008) Cost effectiveness analysis of the emission abatement in the shipping sector emissions, JRC scientific and technical reports. | | | yes |

| | | | | | |
|---|---|---|---|---|---|
| 0 | | Back, S., Ekebom, J., Kangas, J. (2002) A proposal for a long-term baseline phytobenthos monitoring programme for the Finnish Baltic coastal waters: Monitoring submerged rocky shore vegetation, Environmental Monitoring and Assessment 79(1):13-27. | yes | | |
| 0 | | Balana, B. B., Jackson-Blake, L., Martin-Ortega, J., & Dunn, S. (2015) Integrated cost-effectiveness analysis of agri-environmental measures for water quality, Journal of Environmental Management 161: 163-172. | | | yes |
| 0 | | Balana, B. B., Vinten, A., & Slee, B. (2011) A review on cost-effectiveness analysis of agri-environmental measures related to the EU WFD: Key issues, methods, and applications, Ecological Economics 70(6):1021-2031. | yes | yes | yes |
| 1 | 0 | Balana, B., Kontolaimou, A., Psaltopoulos, D., Tainio, A., Varjopuro, R. (2014) Pan-European review of cost-effectiveness analysis studies relating to water quality and Directive compliance challenges, Deliverable 6.10 Adaptive strategies to mitigate the impacts of climate change on European freshwater ecosystems REFRESH. | | | yes |
| 0 | | Bastardie, F., Danto, J., Rufener, M-C., van Denderen, D., Eigaard, O.R., Dinesen, G.E., Nielsen, J.R. (2020) Reducing fisheries impacts on the seafloor: A bio-economic evaluation of policy strategies for improving sustainability in the Baltic Sea, Fisheries research 230 . | yes | yes | |
| 0 | | Beisiegel, K., Darr, A., Gogina, M., Zettler, M-L. (2017) Benefits and shortcomings of non-destructive benthic imagery for monitoring hard-bottom habitats, Marine Pollution Bulletin 121(1-2): 5-15. | | yes | |

| | | | | | |
|---|---|---|---|---|---|
| 0 | | Berezina, N.A., Gubelit, Y.I., Polyak, Y.M., Sharov, A.N., Kudryavtseva, V.A., Lubimtsev, V.A., Petukhov, V.A., Shigaeva, T.D. (2017) An integrated approach to the assessment of the eastern Gulf of Finland health: A case study of coastal habitats, Journal of Marine Systems 171: 159-171. | | yes | |
| 1 | 0 | Bertram, C., Rehdanz, K. (2013) On the environmental effectiveness of the EU marine strategy framework directive, Marine Policy 38: 25-40. | yes | | |
| 0 | | Bouraoui, F., & Grizzetti, B. (2014) Modelling mitigation options to reduce diffuse nitrogen water pollution from agriculture, Science of The Total Environment 468-469: 1267-1277. | | | yes |
| 1 | 0 | Brady, M. (2004) Managing agriculture and water quality: Four essays on the control of large-scale nitrogen pollution, PhD thesis. | | | yes |
| 1 | 0 | Brady, M. (2003) The relative cost-efficiency of arable nitrogen management in Sweden, Ecological Economics 47(1): 53-70. | yes | yes | yes |
| 0 | | Brady, M. (2002) The cost-efficiency of Swedish arable-nitrogen abatement policy, conference contribution. | | | yes |
| 0 | | Brady, M. (2002) Stochastic arable-nitrogen abatement from heterogeneous sources - cost-effective coastal nitrogen abatement, conference contribution. | | | yes |
| 1 | 0 | Brady, M.V., Hristov, J., Wilhelmsson, F., Hedlund, K. (2019) Roadmap for Valuing Soil Ecosystem Services to Inform Multi-Level Decision-Making in Agriculture, SUSTAINABILITY 11(19). | yes | | |

| | | | | | |
|---|---|---|---|---|---|
| 0 | | Bratli, J. L., Svelle, M., Ibrekk, H. O.  (1995)  Norwegian North Sea action programme: Analysis of measures to reduce nutrient inputs, Coastal Management 23(4): 241-263. | | | yes |
| 0 | | Brodersen, S.L., Konrad, M.T.H., Hasler, B., Munch, K. (2009) The Costs of a Cleaner Baltic Sea, conference abstract. | | | yes |
| 1 | 1 | Bryhn, A. C.  (2009)  Sustainable Phosphorus Loadings from Effective and Cost-Effective Phosphorus Management Around the Baltic Sea, PloS one 4(5). | | | yes |
| 0 | | Bryhn, A. C., & Håkanson, L. (2009)  Coastal eutrophication: Whether N and/or P should be abated depends on the dynamic mass balance, Proceedings of the National Academy of Sciences 106(1). | | | yes |
| 0 | | Buch, E., Elken, J., Gajewski, J., Haakansson, B., Kahma, K., Soetje, K. (2004) Baltic operational oceanographic system - BOOS, conference paper. | | yes | |
| 1 | 0 | Byström, O. (1996) Costs and values of wetlands for nitrogen abatement in Sweden, thesis. | | | yes |
| 1 | 0 | Byström, O. (1998) Cost-effective reduction of nonpoint source pollution: five essays on wetlands, policy and modeling, PhD thesis. | | | yes |
| 1 | 0 | Byström, O.  (1998)  The nitrogen abatement cost in wetlands, Ecological Economics 26(3): 321-331. | | | yes |
| 1 | 0 | Byström, O.  (2000)  The Replacement Value of Wetlands in Sweden, Environmental and Resource Economics 16(4): 347-362. | yes | yes | yes |

| | | | | | |
|---|---|---|---|---|---|
| 0 | | Bäck, S., Ekebom, J., Kangas, P. (2002) A proposal for a long-term baseline phytobenthos monitoring programme for the finnish baltic coastal waters: monitoring submerged rocky shore vegetation, Environmental monitoring and assessment 79(1): 13-27. | | yes | |
| 1 | 0 | Carolus, J.F., Bartosova, A., Olse, S.B., Jomaa, A. (2020) Nutrient mitigation under the impact of climate and land-use changes: a hydro-economic approach to participatory catchment management, Journal of Environmental management 271(1). | yes | yes | yes |
| 0 | | Carolus, J.F., Bartsova, A., Pedersen, S.M., Olsen, S.B., Jomaa, S., Veinbergs, A. (2018) Cost-effectiveness analysis of nutrient mitigating measures: a cross-country comparison under the impact of climate change, conference poster. | | | yes |
| 0 | | Collentine, D. Program design for a tradable permit system for nutrient loads to the Baltic Sea, [quote] | | | yes |
| 1 | 0 | Collentine, D., & Johnsson, H. (2012) Crop Discharge Permits for Reduction of Nitrogen Loads to the Baltic Sea1, JAWRA Journal of the American Water Resources Association 48(1):24-31. | | | yes |
| 1 | 0 | Collentine, D., & Johnsson, H. (2013) Evaluating the effect of climate variation on the cost efficiency of a crop permit policy in Southern Sweden, Journal of Water and Climate Change 4(2): 110-117. | yes | yes | yes |

| | | | | | |
|---|---|---|---|---|---|
| 0 | | Collentine, D., Eckersten, H., Norman Haldén, A., Ryd Ottoson, J., Salomon, E., Sundin, S., Tattari, S., Braun, J., Kuussaari, M. (2013) Consequences of future nutrient load scenarios on multiple benefits of agricultural production, Report from the Department of Crop Production Ecology (VPE) (1653-5375), SLU. | | | yes |
| 0 | | Collentine, D., Johnsson, H., Larsson, P., Markensten, H., Widén Nilsson, E. (2012) Development of cost effective nutrient management strategies for a watershed with the DSS FyrisCOST, conference abstract. | | | yes |
| 0 | | Collins, A. L., Newell Price, J. P., Zhang, Y., Gooday, R., Naden, P. S., & Skirvin, D. (2018) Assessing the potential impacts of a revised set of on-farm nutrient and sediment 'basic' control measures for reducing agricultural diffuse pollution across England, Science of The Total Environment 621: 1499-1511. | | | yes |
| 1 | 1 | Czajkowski, M., Andersen, H.E., Blicher-Mathiasen, G., Budzinski, W., Elofsson, K., Hagemejer, J., Hasler, B., Humborg, C., Smart, J.C.R., Smedberg, E., Stålnacke, P., Was, A., Wilamowski, M., Zylicz, T., Hanley, N. (2019) Increasing the cost-effectiveness of water quality improvements through pollution abatement target-setting at different spatial scales, Discussion papers in environmental and one health economics, paper no 2019-05, University of Glasgow. | yes | | yes |
| 1 | 0 | Dalgaard, T., Hansen, B., Hasler, B., Hertel, O., Hutchings, N. J., Jacobsen, B. H., . . . Vejre, H. (2014) Policies for agricultural nitrogen management—trends, challenges and prospects for improved efficiency in Denmark, Environmental Research Letters 9(11). | | | yes |

| | | | | | |
|---|---|---|---|---|---|
| 0 | | de Haan, J., van der Schoot, J. R., Verstegen, H., & Clevering, O. (2010) Removal of nitrogen leaching from vegetable crops in constructed wetlands, ISHS Acta Horticulturae 852. | | | yes |
| 0 | | Destandau, F., Martin, E., Rozan, A. (2011) Potential of artificial wetlands for removing pesticides from water in a cost-effectiveness framework, paper prepared for presentation at the EAAE congress 2011. | | | yes |
| 0 | | Destandau, F., Martin, E., Rozan, A. Potential of artificial wetlands for removing pesticides from water in a cost-effectiveness framework, [quote], paper on the list above | | | yes |
| 0 | | Eichler, F., & Schulz, D. (1998) The nitrogen reduction programme in the Federal Republic of Germany, Environmental Pollution 102(1): 609-617. | | | yes |
| 1 | 0 | Elmgren, R., & Larsson, U. (2001) Nitrogen and the Baltic Sea: Managing Nitrogen in Relation to Phosphorus, The Scientific World JOURNAL 1. | | | yes |
| 1 | 0 | Elofsson, K. (2002) Economics of marine pollutions, PhD thesis. | | | yes |
| 0 | | Elofsson, K. (2003) Control strategies for interactive water pollutants, conference paper. | | | yes |
| 1 | 1 | Elofsson, K. (2010) Cost-effectiveness of the Baltic Sea action plan, Marine Policy 34(5): 1043-1050. | yes | yes | yes |
| 1 | 1 | Elofsson, K. (2014) International knowledge diffusion and its impact on the cost-effective clean-up of the Baltic Sea, SLU working paper. | | | yes |
| 0 | | Elofsson, K. Cost-effectiveness of the Baltic Sea Action Program, [quote] | | | yes |

| | | | | | |
|---|---|---|---|---|---|
| 1 | 1 | Elofsson, K. (1999) Cost Effective Reductions in the Agricultural Load of Nitrogen to the Baltic Sea, In M. Boman, R. Brännlund & B. Kriström (Eds.) Topics in Environmental Economics. Economy and Environment. 17. | | | yes |
| 1 | 1 | Elofsson, K. (2003) Cost-effective reductions of stochastic agricultural loads to the Baltic Sea, Ecological Economics 47(1): 13-31. | yes | yes | yes |
| 1 | 0 | Elofsson, K. (2006) Cost-Effective Control of Interdependent Water Pollutants, Environmental Management 37(1): 54-68. | yes | yes | yes |
| 1 | 1 | Elofsson, K. (2010) The Costs of Meeting the Environmental Objectives for the Baltic Sea: A Review of the Literature, AMBIO 39(1): 49-58. | | | yes |
| 1 | 0 | Elofsson, K. (2012) Swedish nutrient reduction policies: an evaluation of cost-effectiveness, Regional Environmental Change 12(1): 225-235. | yes | yes | yes |
| 0 | | Elofsson, K., & von Brömssen, C. (2017) The revealed preferences of Baltic Sea governments: Goals, policy instruments, and implementation of nutrient abatement measures, Marine Pollution Bulletin 118:188-196. | | | yes |
| 0 | | Elofsson, K., Folmer, H., Gren, I.-M. (2003) Management of eutrophicated coastal ecosystems: a synopsis of the literature with emphasis on theory and methodology, Ecological Economics 47(1): 1-11. | | | yes |
| 1 | 1 | Elofsson, K. (2010) Baltic-wide and Swedish nutrient reduction targets - an evaluation of cost-effective strategies, Report to the expert group for environmental studies 2010:2, Ministry of Finance, Sweden. | | | yes |

| | | | | | |
|---|---|---|---|---|---|
| 0 | | Elofsson, K., Iho, A., Ahlvik, L. (2016) Literature review and learning from previous experiences on emissions and nutrient discharge trading outside the BSR, NutriTrade report. | | | yes |
| 0 | | Eory, V., Topp, C. F. E., & Moran, D. (2013) Multiple-pollutant cost-effectiveness of greenhouse gas mitigation measures in the UK agriculture, Environmental Science & Policy 27: 55-67. | | | yes |
| 0 | | Ericsson, B., & Hallmans, B. (1994) Control of the nutrient pollution discharge from the Vistula River Basin in Poland, Desalination 98(1): 185-197. | | | yes |
| 0 | | Feiz, R., Ammenberg, J. (2017) Assessment of feedstocks for biogas production, part I-A multi-criteria approach, Resources conservation and recycling 122. | yes | yes | |
| 0 | | Ferreira, J. G., & Bricker, S. B. (2019) Assesment of nutrient trading services from bivalve farming,In A. C. Smaal, J. Ferreira, J. Grant, J. Petersen, & Ø. Strand (Eds.) Goods and services from bivalve farming. | | | yes |
| 0 | | Fröschl, L., Pierrard, R., & Schönbäck, W. (2008) Cost-efficient choice of measures in agriculture to reduce the nitrogen load flowing from the Danube River into the Black Sea: An analysis for Austria, Bulgaria, Hungary and Romania, Ecological Economics 68(1):96-105 | | | yes |
| 1 | 0 | Gachango, F. G., Pedersen, S. M., & Kjaergaard, C. (2015) Cost-Effectiveness Analysis of Surface Flow Constructed Wetlands (SFCW) for Nutrient Reduction in Drainage Discharge from Agricultural Fields in Denmark, Environmental Management 56(6): 1478-1486. | | | yes |

| | | | | | |
|---|---|---|---|---|---|
| 1 | 1 | Georgiou S., Turner R.K., Bateman I.J. (2001) Valuation and Management of Nutrient Pollution in the Baltic Drainage Basin, In: Turner R.K., Bateman I.J., Adger W.N. (eds.) Economics of Coastal and Water Resources: Valuing Environmental Functions. Studies in Ecological Economics 3. | | | yes |
| 0 | | Gren I.-M., Brännlund, R. (1995) Enforcement of regional environmental regulations: nitrogen fertilizers in Sweden, In: Hanna, S., Munasinghe, M. (eds.) Property rights in a social and ecological context: case studies and design application vol. 994. | | | yes |
| 1 | 0 | Gren, I.-M. (1993) Alternative nitrogen reduction policies in the Mälar region, Sweden, Ecological Economics 7(2): 159-172. | | | yes |
| 1 | 0 | Gren, I.-M. (1995) Costs and benefits of restoring wetlands: two Swedish case studies, Ecological Engineering 4(2): 153-162. | | | yes |
| 1 | 1 | Gren, I.-M. (2008) Adaptation and mitigation strategies for controlling stochastic water pollution: An application to the Baltic Sea, Ecological Economics 66(2): 337-347. | yes | yes | |
| 1 | 0 | Gren, I.-M. (2010) Climate change and the Water Framework Directive: cost effectiveness and policy design for water management in the Swedish Mälar region, Climatic Change 100(3): 463-484. | | | yes |
| 1 | 1 | Gren, I.-M. (2017) Costs of nutrient management with technological development and climate change, In R. Bali Swain (Ed.) Environmental challenges in the Baltic Region | | | yes |
| 1 | 0 | Gren, I.-M. (2019) The economic value of mussel farming for uncertain nutrient removal in the Baltic Sea, PloS one 14(6) . | | | yes |

| | | | | | |
|---|---|---|---|---|---|
| 1 | 1 | Gren, I.-M., & Ang, F. (2019) Stacking of abatement credits for cost-effective achievement of climate and water targets, Ecological Economics 164, 106375. | yes | yes | yes |
| 1 | 1 | Gren, I.-M., & Destouni, G. (2012) Does Divergence of Nutrient Load Measurements Matter for Successful Mitigation of Marine Eutrophication? AMBIO 41(2): 151-160 | yes | yes | yes |
| 1 | 0 | Gren, I.-M., & Elofsson, K. (2017) Credit stacking in nutrient trading markets for the Baltic Sea, Marine Policy 79: 1-7. | | | yes |
| 1 | 1 | Gren, I.-M., & Säll, S. (2015) Cost-effective nutrient and green-house gas management in the Baltic Sea region, Environmental economics 6(1): 80-90. | | | yes |
| 1 | 0 | Gren, I.-M., Lindahl, O., & Lindqvist, M. (2009) Values of mussel farming for combating eutrophication: An application to the Baltic Sea, Ecological Engineering 35(5): 935-945. | yes | yes | yes |
| 0 | | Gren, I.-M., Nyström Sandman, A., & Näslund, J. (2018) Aquatic invasive species and ecosystem services: Economic effects of the worm Marenzelleria spp in the Baltic Sea, Water Resources and Economics 24: 13-24. | yes | yes | yes |
| 1 | 1 | Gren, I.-M., Savchuk, O. P., Jansson, T. (2013) Cost-Effective Spatial and Dynamic Management of a Eutrophied Baltic Sea, Marine Resource Economics 28(3): 263-284. | yes | yes | yes |
| 1 | 0 | Gren, I.-M., Säll, S., Zeleke Akilu, A., & Tirkaso, W. (2018) Does mussel farming promote cost savings and equity in reaching nutrient targets for the Baltic Sea? Water 10(11). | yes | yes | yes |
| 1 | 1 | Gren, I.-M., Söderqvist, T., & Wulff, F. (1997) Nutrient Reductions to the Baltic Sea: Ecology, Costs and Benefits, Journal of Environmental Management 51(2): 123-143. | | | yes |

| | | | | | |
|---|---|---|---|---|---|
| 1 | 1 | Gren, I.-M. (1999) Value of land as a pollutant sink for international waters,Econological economics 30(3): 419-431. | yes | yes | |
| 1 | 0 | Gren, I.-M. (2015) Estimating values of carbon sequestration and nutrient recycling in forests; an application to the Stockholm-Mälar region in Sweden,Forests 6: 3594-3613. | | yes | yes |
| 1 | 1 | Gren, I.-M. (2017) Cost-effective nutrient reductions to the Baltic Sea, Managing a sea: the ecological economics of the Baltic: 43-56. | | yes | |
| 0 | | Gren, I.-M. (2009) A numerical model for dynamic cost effective mitigation of eutrophication with spatial heterogeneity in the Baltic Sea, SLU Working paper. | | | yes |
| 0 | | Gren, I.-M., Elofsson, E. (2013) Market power and double-dipping in nutrient trading markets, SLU working paper. | | | yes |
| 1 | 1 | Gren, I.-M., Jannke, P., Elofsson, K. (1997) Cost-effective nutrient reductions to the Baltic Sea, Environmental and resource economics 10(341-362). | | yes | |
| 1 | 1 | Gren, I.-M., Jonzon, Y., Lindqvist, M. (2008) Costs of nutrient reduction to the Baltic Sea, SLU working paper. | | | yes |
| duplicate | | Gren, I.-M., Jonzon, Y., Lindqvist, M. (2008) Cost of nutrient reductions to the Baltic Sea, SLU Working paper. | | | yes |
| 0 | | Gren, I.-M., Sandman Nyström, A., Näslund, J. (2016) Economic effects of the invasive worm Marenzelleria spp. in the Baltic Sea, working paper 2016:11, Department of economics, SLU. | | | yes |
| 0 | | Gren, I.-M., Turner, R.K., Wulff, F. (2017) Winners and losers from Baltic Sea nitrogen reductions, [quote] book: Managing a sea. | | | yes |

| | | | | | |
|---|---|---|---|---|---|
| 1 | 0 | Grossmann, M. (2012) Economic value of the nutrient retention function of restored floodplain wetlands in the Elbe River basin, Ecological Economics 83: 108-117. | | | yes |
| 1 | 1 | Halkos, G.E., & Galani, G. K. (2016) Cost-Effectiveness Analysis in Reducing Nutrient Loading in Baltic and Black Seas: A Review, Journal of Environmental Management and Tourism 5(1): 28-51. | | yes | yes |
| 1 | 0 | Hart, R., & Brady, M. (2002) Nitrogen in the Baltic Sea—policy implications of stock effects, Journal of Environmental Management 66(1): 91-103. | yes | yes | yes |
| 0 | | Hasler, B., Czajkowski, M., Elofsson, K., Hansen, L. B., Konrad, M. T., Nielsen, H. Ø., . . . Zagórska, K. (2019) Farmers' preferences for nutrient and climate-related agri-environmental schemes: A cross-country comparison, AMBIO 48(11): 1290-1303. | | | yes |
| 0 | | Hasler, B., Hyytiäinen, K., Refsgaard, J.C., Smart, J.C.R., & Tonderski, K. (2019) Sustainable ecosystem governance under changing climate and land use: An introduction, AMBIO 48(11): 1235-1239. | | | yes |
| 1 | 1 | Hasler, B., Smart, J.C.R., Fonnesbech-Wulff, A., Andersen, H.E., Thodsen, H., Blicher Mathiesen, G., . . . Wulff, F. (2014) Hydro-economic modelling of cost-effective transboundary water quality management in the Baltic Sea, Water Resources and Economics 5: 1-23. | | | yes |
| 0 | | Hasler, B., Smart, J.C.R., Fonnesbech-Wulff, A., Andersen H.E., Thodsen, H., Blicher-Mathiesen, G. (2012) Regional cost-effectiveness in transboundary water quality management for the Baltic Sea, Conference paper. | | | yes |

| | | | | | |
|---|---|---|---|---|---|
| 0 | | Hasler, B., Block Hansen, L., Konrad, M., Termansen, M., Andersen, H.E. (2014) Cost-efficient targeted location of N reduction measures by spatial hydro-economic modelling. | | | yes |
| 0 | | Hasler, B., Czajkowski, M., Elofsson, K., Smart, J.C.R., Waas, A., Konkrad, M., Andersen, H.E., Thodsen, H., Göke, C., Mörth, M., Smedberg, E., Humborg, C. (2011) Integrated modelling to support cost-effective management of nutrient reductions to the Baltic Sea, conference abstract. | | | yes |
| 0 | | Hasler, B., Neye, S., Shou J.S., Martinsen, L. (2007) Modelling cost-minimising strategies for improving the aquatic environment of the Baltic sea, Workshop presentation. | | | yes |
| 0 | | Hasler, B., Smart, J.C.R., Fonnesbech-Wulff, A., Andersen, H.E., Thodsen, H., Blicher Mathiesen, G., Smedberg, E., Göke, C., Czajkowski, M., Was, A. ,Elofsson, K., Humborg, C., Wolfsberg, A., Wulff, F. (2014) Water resources and economics, link to Hasler et al. (2014) | | | yes |
| 0 | | Hasler, B., Smart, J.C.R., Fonnesbech-Wulff, A. (2012) Deliverable 8.1,[quote] Deliverable 8.1 | | | yes |
| 1 | 0 | Hautakangas, S., & Ollikainen, M. (2019) Nutrient Trading Between Wastewater Treatment Plants in the Baltic Sea Region, Environmental and Resource Economics 73(2): 533-556. | yes | yes | yes |
| 1 | 1 | Hautakangas, S., Ollikainen, M., Aarnos, K., & Rantanen, P. (2014) Nutrient Abatement Potential and Abatement Costs of Waste Water Treatment Plants in the Baltic Sea Region, AMBIO 43(3): 352-360. | | | yes |

| | | | | | |
|---|---|---|---|---|---|
| 1 | 0 | Hautakangas, S. (2020) Cost-efficient nutrient load reduction in wastewater treatment plants, PhD thesis. | | | yes |
| 0 | | Heeb, A., Johansson, L. (2013) Buffer zones & wetlands as agri-environmental measures in the Baltic Sea region, presentation. | | | yes |
| 0 | | HELCOM (2010) Ecosystem health of the Baltic Sea: HELCOM initial holistic assessment, Baltic Sea Environment Proceedings 122: 1-63. | yes | | |
| 1 | 0 | Helin, J. (2020) Developing improved methods for identifying the cost-efficient abatement set in coastal water quality protection, Journal of Environmental Management 273. | yes | yes | |
| 1 | 0 | Helin, J., & Tattari, S. (2012) How much can be gained by optimizing nutrient abatement spatially – Cost-efficiency comparison of non-point arable loads from different Finnish watersheds, Food Economics 9(1-2): 95-107. | | | yes |
| 1 | 0 | Helin, J. (2013) Cost efficient nutrient load reduction in agriculture. A short-run perspective on reducing nitrogen and phosphorus in Finland, PhD thesis: MTT Science. | | | yes |
| 0 | | Helleman, K., Zachai, R. (1999) Recent progress in mm-wave-sensor system capabilities for enhances (synthetic) vision, Proceedings of SPIE - The International Society for Optical Engineering 3691: 21-28. | yes | yes | |
| 0 | | Honkatukia, J., Ollikainen, M. (2001) Towards efficient pollution control in the Baltic Sea: an anatomy of current failure with suggestions, ETLA Discussion Papers 755. | | | yes |
| duplicate | | Honkatukia, J., Ollikainen, M. Towards efficient pollution control in the Baltic Sea,ETLA Discussion Papers 755. | | | yes |
| 0 | | Hürdler, J., Venohr, M. Monthly nutrient emissions and loads to the Odra river basin, poster | | | yes |

| | | | | | |
|---|---|---|---|---|---|
| 1 | 0 | Hyytiainen, K., Ahtiainen, H., Heikkila, J., Helin, J., Huhtala, A., Iho, A., . . . Vesterinen, J. (2009) An integrated simulation model to evaluate national policies for the abatement of agricultural nutrients in the Baltic Sea, Agricultural and Food Science 18(3-4): 440-459. | | | yes |
| 1 | 1 | Hyytiäinen, K., & Ahlvik, L. (2015) Prospects for cost-efficient water protection in the Baltic Sea, Marine Pollution Bulletin 90(1): 188-195. | | yes | yes |
| duplicate | | Hyytiäinen, K., & Ahlvik, L. (2015) Prospects for cost-efficient water protection in the Baltic Sea, Marine Pollution Bulletin 90(1): 188-195. | | | yes |
| 1 | 1 | Hyytiäinen, K., Ahlvik, L., Ahtiainen, H., Artell, J., Huhtala, A., & Dahlbo, K. (2015) Policy Goals for Improved Water Quality in the Baltic Sea: When do the Benefits Outweigh the Costs? Environmental and Resource Economics 61(2): 217-241. | yes | yes | yes |
| duplicate | | Hyytiäinen, K., Ahtiainen, H., & Heikkilä, J. (2009) An integrated simulation model to evaluate national measures for the abatement of agricultural nutrients in the Baltic Sea, Agricultural and Food Science 18(3-4). | | | yes |
| 0 | | Hyytiäinen, K., Hasler, B., Ericsdotter, S., Nekoro, M., Blyh, K., Artell, J., Ahlvik, L., Ahtiainen, H. (2013) Worth it: benefits outweigh costs in reducing eutrophication in the Baltic, BalticSTERN summary report for HELCOM 2013 ministerial meeting. | | | yes |
| 0 | | Hyytiäinen, K., Ahlvik, L., Ahtiainen, H., Artell, J., Dahlbo, K., Huhtala, A. (2013) Spatially explicit bio-economic modelling for the Baltic Sea: do the benefits outweigh the costs? MTT discussion papers 2, 2013. | | | yes |

| | | | | | |
|---|---|---|---|---|---|
| 1 | 1 | Hyytiäinen, K., Blyh, K., Hasler, B., Ahlvik, L., Ahtiainen, H., Artell, J., Ericsdotter, S. (2014) Environmental economic research as a tool in the protection of the Baltic Sea - costs and benefits of reducing eutrophication, TemaNord 2014:504. | | | yes |
| 0 | | Håkanson, L., & Bryhn, A. C.  (2010)  Controlling Eutrophication in the Baltic Sea and the Kattegat,In: Ansari A., Singh Gill S., Lanza G., Rast W. (eds) Eutrophication: causes, consequences and control. | yes | yes | yes |
| 0 | | Håkanson, L., Stabo, H. R., & Bryhn, A. C.  (2010)  Strategies for Remediation, Cost-Benefit Analyses and a Holistic Management Plan for the Baltic Sea. ,In: The Fish Production Potential of the Baltic Sea. Environmental Science and Engineering. | | | yes |
| 0 | | Håkanson, L., Bryhn, A.C. (2008) Empirical data and models on nutrients and bioindicators in the Baltic Sea. | | | yes |
| 1 | 0 | Håkansson, L. (2009) Modeling nutrient fluxes to, within and from the Kattegat to find an optimal, cost-efficient Swedish remedial strategy. | | | yes |
| 0 | | Häggmark Svensson, T. (2019) Essays on water quality technologies of the Baltic Sea and technological innovation, PhD thesis SLU. | | | yes |
| 1 | 1 | Häggmark Svensson, T., & Elofsson, K.  (2019)  The Ex-Post Cost-Effectiveness of Nitrogen Load Reductions From Nine Countries to the Baltic Sea Between 1996 and 2010, Water Resources Research 55(6): 5119-5134. | yes | yes | yes |
| 0 | | Iho, A. (2017) Report on possible application areas of nutrient trading in Finnish and Swedish water protection policy. Note author is actually Ek, Claes. | | | yes |

| | | | | | |
|---|---|---|---|---|---|
| 1 | 0 | Iho, A.  (2005)  Does scale matter? Cost-effectiveness of agricultural nutrient abatement when target level varies, Agricultural and Food Science 14(3). | | | yes |
| 0 | | Iho, A., Ribaudo, M., & Hyytiäinen, K.  (2015)  Water protection in the Baltic Sea and the Chesapeake Bay: Institutions, policies and efficiency, Marine Pollution Bulletin 93(1):81-93. | yes | yes | yes |
| 1 | 0 | Jabłońska, E., Wiśniewska, M., Marcinkowski, P., Grygoruk, M., Walton, C. R., Zak, D., . . . Kotowski, W.  (2020)  Catchment-Scale Analysis Reveals High Cost-Effectiveness of Wetland Buffer Zones as a Remedy to Non-Point Nutrient Pollution in North-Eastern Poland, Water 12(3). | | | yes |
| 0 | | Jacobsen, B.H. Marginal cost of reducing nitrogen losses to water and air in Denmark [quote] | | | yes |
| 0 | | Jansson, T., Andersen, H. E., Gustafsson, B. G., Hasler, B., Höglind, L., & Choi, H.  (2019)  Baltic Sea eutrophication status is not improved by the first pillar of the European Union Common Agricultural Policy, Regional Environmental Change 19(8):2465-2476. | | | yes |
| 1 | 0 | Jensen, A. K., Uggeldahl, K. C., Jacobsen, B. H., Jensen, J. D., & Hasler, B.  (2019)  Including aesthetic and recreational values in cost-effectiveness analyses of land use change based nitrogen abatement measures in Denmark, Journal of Environmental Management 240: 384-393. | | | yes |
| 0 | | Jensen-Juul, M. (2019) PhD defense: participatory decision-support tools to improve environmental management- experiences from the Baltic Sea region | | | yes |

| | | | | | |
|---|---|---|---|---|---|
| 0 | | Jetoo, S.  (2018)  Barriers to Effective Eutrophication Governance: A Comparison of the Baltic Sea and North American Great Lakes, Water 10(4):400. | | | yes |
| 1 | 0 | Kageson, P. (1999) Economic isntruments for reducing emissions from sea transport, Air pollution and climate series no. 11 / T&E REPORT 99/7 | | | yes |
| 0 | | Kalli J. (2013) Cost efficiency estimations of in force and forthcoming international regulations (Marpol annex IV),[quote] | | | yes |
| 0 | | Kalli J. (2013) Cost efficiency estimations of abatement techniques,[quote] | | | yes |
| 0 | | Kari, E., Kratzer, S., Beltrán-Abaunza, J.M., Harvey, E.T., Vaicute, D (2017) Retrieval of suspended particulate matter from turbidity-model development, validation, and application to mERIS data over the Baltic Sea, International journal of remote sensing 38(7):1983-2003. | | yes | |
| 0 | | Katila, J. (2013) Environmentally differentiated port fees in the Baltic Sea ports: building a cost-efficient port fee system, conference paper. | | | yes |
| 0 | | Kazmierczak, R.F. Jr, Doering, O., Diaz-Hermelo F., Heimlich, R., Hitzhusen, F., Howard, C., Libby, L., Milon, W., Prato, A., Ribaudo, M. (1999) The downstream implications of nutrient overload: the hypoxia problem in the northern Gulf of Mexico, conference proceedings. | | | yes |

| | | | | | |
|---|---|---|---|---|---|
| 1 | 0 | Kiirikki, M., Rantanen, P., Varjopuro, R., Leppänen, A., Hiltunen, M., Pitkänen, H., Ekholm, P., Moukhametshina, E., Inkala , A., Kuosa, H., Sarkkula, J. (2003) Cost effective water protection in the Gulf of Finland - focus on St. Petersburg, Finnish environment institute, report 632. | | | yes |
| 0 | | Kinnunen, P., Iho, A. (2017) Expert and stakeholder meetings in Finland. First stepping stones, NutriTrade report. | | | yes |
| 0 | | Klõga M., Leal Filho W., Fischer N. (2015) Innovative approaches towards sustainable river basin management in the Baltic Sea Region: The WATERPRAXIS project,In: Leal Filho W., Sümer V. (eds) Sustainable Water Use and Management. Green Energy and Technology. | | | yes |
| 0 | | Konovalenko, L., Bradshaw, C., Andersson, E., Lindqvist, D., Kautsky, U. (2016) Evaluation of factors influencing accumulation of stable Se and Cs in lake and coastal fish, Journal of Environmental Radioactivity 160: 64-79. | | yes | |
| 1 | 0 | Konrad, M.T., Andersen, H.E., Thodsen, H., Termansen, M., & Hasler, B. (2014) Cost-efficient reductions in nutrient loads; identifying optimal spatially specific policy measures, Water Resources and Economics 7(39-54). | | | yes |
| 0 | | Konrad, M., Andersen H.E., Thodsen, H., Termansen, M., Hasler, B. (2012) Cost-efficient reductions in nutrient loads; optimal spatial policy measures to meet water quality targets at multiple locations. | | | yes |

| | | | | | |
|---|---|---|---|---|---|
| 0 | | Kortsch, P., Kirsch, F. (2018) Pile driving of open-ended steel piles in offshore wind industry - comparison of prognosis and measurements, Geotechnik 41(1):30-39. | | yes | |
| 0 | | Koskiaho, J., Okruszko, T., Piniewski, M., Marcinkowski, P., Tattari, S., Johannesdottir, S., . . . Kämäri, M.  (2020)  Carbon and nutrient recycling ecotechnologies in three Baltic Sea river basins – the effectiveness in nutrient load reduction, Ecohydrology & Hydrobiology, | | | yes |
| 0 | | Koskiaho, J., Tattari, S,, Röman, E. (2015) Suspended solids and total phosphorus loads and their spatial differences in a lake-rich river basin as determined by automatic monitoring network, Environmental monitoring and assessment 187(4). | | yes | |
| 1 | 0 | Kotta, J., Futter, M., Kaasik, A., Liversage, K., Rätsep, M., Barboza, F. R., . . . Virtanen, E.  (2020)  Cleaning up seas using blue growth initiatives: Mussel farming for eutrophication control in the Baltic Sea, Science of The Total Environment 709 | yes | yes | yes |
| 0 | | Kratzer S., Ebert K., Sørensen K. (2011) Monitoring the bio-optical state of the Baltic Sea ecosystem with remote sensing and autonomous in situ techniques, In: Harff J., Björck S., Hoth P. (eds) The Baltic Sea Basin. Central and Eastern European Development Studies (CEEDES). | yes | | yes |
| 0 | | Kulmala, S., Ahlvik, L., Varjopuro, R., Eriksson, A. (2012) Knowledge-based sustainable management for Europe's seas, KnowSeas Deliverable 7.2. | | | yes |

| | | | | | |
|---|---|---|---|---|---|
| 0 | | Lancelot, C., Thieu, V., Polard, A., Garnier, J., Billen, G., Hecq, W., & Gypens, N. (2011) Cost assessment and ecological effectiveness of nutrient reduction options for mitigating Phaeocystis colony blooms in the Southern North Sea: An integrated modeling approach, Science of The Total Environment 409(11): 2179-2191. | | | yes |
| 0 | | Lange, B., Larse, S., Hökstrup, J., Barthelmie, R. (2004) Importance of thermal effects and sea surface roughness for offshore wind resource assessment, Journal of wind engineering and industrial aerodynamics 92(11): 959-988. | | yes | |
| 1 | 0 | Lindqvist, M. (2013) Cost effective nutrient abatement in the Baltic Sea, PhD thesis. | | | yes |
| 1 | 1 | Lindqvist, M., Gren, I.-M. (2013) Cost effective nutrient abatement for the Baltic Sea under learning-by-doing induced technical change, SLU Working paper | | | yes |
| duplicate | | Lindqvist, Martin, Gren, I.-M. (2013) Cost effective nutrient abatement under learning-by-doing induced technical change, SLU Working paper. | | | yes |
| 0 | | Lundberg, C. (2005) Conceptualizing the Baltic Sea Ecosystem: An Interdisciplinary Tool for Environmental Decision Making, AMBIO: A Journal of the Human Environment 34(6433-439). | | | yes |
| 1 | 0 | Lötjönen, S., & Ollikainen, M. (2019) Multiple-pollutant cost-efficiency: Coherent water and climate policy for agriculture, AMBIO 48(11):1304-1313. | yes | | yes |
| 0 | | Mander, Ü., & Meyer, B. C. (2012) Adaptation and functional water management through land use change, Ecological Indicators 22. | | | yes |

| | | | | | |
|---|---|---|---|---|---|
| 1 | 0 | Markowska, A., Zylicz, T. (1999) Costing an international public good; the case of the Baltic Sea, Ecological Economics 30(2):301-316. | yes | yes | |
| 0 | | Martinez, M., Esteve, M.A., Martínez-Pz, J.M., Carreño, F., Robledano, F., Ruiz, M., Alonso, F. (2007) Simulating management options and scenarios to control nutrient load to Mar Menor, Southeast Spain, Transitional waters monograph 1:53-70. | | | yes |
| 0 | | Martin-Ortega, J., Balana, B.B. (2012) Cost-effectiveness analysis in the implementation of the water framework directive: a comparative analysis of the United Kingdom and Spain, European Water 37:15-25. | | | yes |
| 0 | | Mayerle, R., Schroeter, A., Zielke, W. (1995) Simulation of nearshore wave current interaction by coupling a Boussinesq wave model with a 3d hydrodynamic model, conference paper. | | yes | |
| 0 | | Meulen, N.V. (2015) A cost effectiveness analysis using wetland treatment systems and sediment ponds to reduce excess nutrients in the Maumee River Watershed, Bachelor's thesis. | | | yes |
| 0 | | Mewes, M. (2007) Cost-effectiveness analysis to prevent nutrient emissions by diffuse sources-Baltic Sea catchment area of Germany,[quote] | | | yes |
| 1 | 0 | Mewes, M. (2012) Diffuse nutrient reduction in the German Baltic Sea catchment: Cost-effectiveness analysis of water protection measures, Ecological Indicators 22: 16-26. | yes | yes | yes |

| | | | | | |
|---|---|---|---|---|---|
| 1 | 0 | Michalak, I., Wilk, R., Chojnacka, K. (2017) Bioconversion of Baltic Seaweeds into Organic Compost,Waste and Biomass Valorization 8(6):1885. | | yes | |
| 1 | 1 | Nainggolan, D., Hasler, B., Andersen, H. E., Gyldenkærne, S., & Termansen, M. (2018) Water Quality Management and Climate Change Mitigation: Cost-effectiveness of Joint Implementation in the Baltic Sea Region, Ecological Economics 144: 12-26. | yes | yes | yes |
| 0 | | Nainngolan, D., Hasler, B., Andersen, H.E., Gyldenkærne, S., Termansen, M. (2014) Paper on effects of socioeconomic scenarios on nutrient loading, GHG emissions and soil organic carbon (manuscript), Deliverable 1.4 BONUS Go4Baltic. | | | yes |
| 0 | | Neumann, T., Schernewski, G. (2001) Cost-effective versus proportional nutrient load reductions to the Baltic Sea: Spatial impact analysis with a 3D-ecosystem model, conference paper. | | yes | |
| 1 | 0 | Neumann, T., Schernewski, G. (2005) An ecological model evaluation of two nutrient abatement strategies for the Baltic Sea, Journal of Marine Systems 56(1-2): 195-206. | | yes | |
| 1 | 0 | Nieminen, E., Ahtiainen, H., Lagerkvist C.-J., Oinonen, S. (2019) The economic benefits of achieving Good Environmental Status in the Finnish marine waters of the Baltic Sea, Marine Policy 99: 181-189. | yes | | |
| 1 | 0 | Nikopoulou, Z (2017) Incremental costs for reduction of air pollution from ships: A case study on North European emission control area, Maritime Policy and Management 44(8): .1056-1077. | | yes | |

| | | | | | |
|---|---|---|---|---|---|
| 1 | 0 | Oinonen, S., Hyytiäinen, K., Ahlvik, L., Laamanen, M., Lehtoranta, V., Salojärvi, J., Virtanen, J. (2016) Cost-effective marine protection - a pragmatic approach, PLoS ONE 11(1). | yes | yes | |
| duplicate | | Oinonen, S., Hyytiäinen, K., Ahlvik, L., Laamanen, M., Lehtoranta, V., Salojärvi, J., Virtanen, J. (2016) Cost-effective marine protection - a pragmatic approach, PLoS ONE 11(1). | yes | yes | |
| 0 | | Ollikainen, M., Hasler, B., Elofsson, K., Iho, A., Andersen, H. E., Czajkowski, M., & Peterson, K. (2019) Toward the Baltic Sea Socioeconomic Action Plan, AMBIO 48(11):1377-1388. | yes | yes | yes |
| 1 | 1 | Ollikainen, M., Honkatukia, J. (2001) Towards efficient pollution control in the Baltic Sea: an anatomy of current failure with suggestions for change, Ambio 30(4): 245-253. | yes | yes | yes |
| 1 | 0 | Owenius, S., Van der Nat, D. (2011) Measures for water protection and nutrient reduction, report Baltic COMPASS. | | | yes |
| 0 | | Pachel, K., Klõga, M., & Iital, A. (2012) Scenarios for reduction of nutrient load from point sources in Estonia, Hydrology Research 43(4):374-382. | | | yes |
| 1 | 0 | Paludan, C., Alexeyev, F. E., Drews, H., Fleischer, S., Fuglsang, A., Kindt, T., . . . Wolter, K. (2002) Wetland management to reduce Baltic Sea eutrophication, Water Science and Technology 45(9): 87-94. | | | yes |
| 0 | | Perttilä, M., Ehlin, U. (1995) The year of the Gulf of Bothnia - experiences and results of a bilateral study programme, European water pollution control 5(3): 14-19. | | yes | |

| | | | | | |
|---|---|---|---|---|---|
| 1 | 0 | Petersen, J. D., Rask, N., Madsen, H. B., Jørgensen, O. T., Petersen, S. E., Nielsen, S. V. K., . . . Jensen, M. H. (2009) Odense Pilot River Basin: implementation of the EU Water Framework Directive in a shallow eutrophic estuary (Odense Fjord, Denmark) and its upstream catchment,In J. H. Andersen & D. J. Conley (Eds.) Eutrophication in Coastal Ecosystems. Developments in Hydrobiology 207. | | | yes |
| 1 | 0 | Petersen, J. K., Hasler, B., Timmermann, K., Nielsen, P., Tørring, D. B., Larsen, M. M., & Holmer, M. (2014) Mussels as a tool for mitigation of nutrients in the marine environment, Marine Pollution Bulletin 82(1):137-143. | | | yes |
| 0 | | Petersen, J. K., Holmer, M., Termansen, M., & Hasler, B. (2019) Nutrient extraction through bivalves,In A. C. Smaal, J. G. Ferreira, G. Jon, J. K. Petersen, & S. Øivind (Eds.), Goods and services of marine bivalves. | | | yes |
| 0 | | Pettersson, A. (2016) Can mussel farming be used as an offset in mitigating eutrophication in the Baltic Sea? Master's thesis, SLU. | | | yes |
| 0 | | Pihlainen, S., Zandersen, M., Hyytiäinen, K., Andersen, H. E., Bartosova, A., Gustafsson, B., . . . Thodsen, H. (2020) Impacts of changing society and climate on nutrient loading to the Baltic Sea, Science of The Total Environment 731. | | | yes |
| 0 | | Pihlajamäki, M., & Tynkkynen, N. (2011) The Challenge of Bridging Science and Policy in the Baltic Sea Eutrophication Governance in Finland: The perspective of Science, AMBIO 40(2): 191-199. | | | yes |

| | | | | | |
|---|---|---|---|---|---|
| 1 | 0 | Pitkänen, H., Bendtsen, J., Hansen, J.L.S., Lehtoranta, J. (2013) Controlling benthic release of phosphorus in different Baltic Sea scales: final report on the result of the PROPPEN project, report . | | | yes |
| 0 | | Quwsar, M.A. (2007) An Economic Analysis of Transparency Improvement in the Baltic Proper, Baltic Sea, Master's thesis, Linköping university. | | | yes |
| 0 | | Ramilan, T., Scrimgeour, F. G. (2006) Abatement Cost Heterogeneity and its Impact on Tradable Nitrogen Discharge Permits, Conference Paper. | | | yes |
| 0 | | Reese, S., Markau, H.J. (2002) Risk handling & natural hazards: new strategies in coastal defense - a case study from Schleswig-Holstein, Germany, conference paper | | yes | |
| 0 | | Reusch, T. B. H., Dierking, J., Andersson, H. C., Bonsdorff, E., Carstensen, J., Casini, M., . . . Zandersen, M. (2018) The Baltic Sea as a time machine for the future coastal ocean, Science Advances 4(5). | | | yes |
| 0 | | Rose, J.M., Bricker, S.B., Tedesco, M.A., Wikfors, G.H. (2014) A role for shellfish aquaculture in coastal nitrogen management, Environmental Science and Technology 48(5):2419-2525. | | | yes |
| 0 | | Rosén, O. (2006) The costs of abatement - a cost-effective allocation of Swedish phosphorous abatement concerning the sea basin of the Baltic proper, Bachelor’s thesis | | | yes |
| 0 | | Savin, A., Strömstedt, E., Leijon, M. (2019) Full-scale measurement of reaction force caused by ice interaction on a buoy connected to a wave energy converter, Journal of Cold Regions Engineering 33(2). | | yes | |

| | | | | | |
|---|---|---|---|---|---|
| 1 | 0 | Scharin, H. (2002) Nutrient management for coastal zones: a case study of the nitrogen load to the Stockholm Archipelago, Water Science and Technology 45(9): 309-315. | yes | yes | yes |
| 0 | | Scharin, H. Net-gains of an Efficient Allocation of Abatement Measures in Improving the Environmental State of Coastal Zones: A study of the nitrogen load to the Stockholm Archipelago. | | | yes |
| 1 | 0 | Schernewski, G., Stybel, N., & Neumann, T. (2012) Zebra Mussel Farming in the Szczecin (Oder) Lagoon: water-quality objectives and cost-effectiveness, Ecology and Society 17(2). | yes | yes | yes |
| duplicate | | Schernewski, G., Stybel, N., & Neumann, T. (2012) Zebra Mussel Farming in the Szczecin (Oder) Lagoon: water-quality objectives and cost-effectiveness, Ecology and Society 17(2). | yes | yes | yes |
| 0 | | Schernewski, G., Stybel, N., Neumann, T. (2012) Managing eutrophication: cost-effectiveness of Zebra mussel farming in the Oder (Szcecin) Lagoon, Workshop presentation. | | | yes |
| 1 | 0 | Schou, J.S., Birr-Pedersen, K. (2006) Cost-effectiveness analysis of measures to reduce nitrogen loads from agriculture: do secondary benefits matter, book: Sustainable irrigation management, technologies and policies. | | | yes |
| 1 | 1 | Schou, J., Neye, S.T., Lundhede, T., Martinsen, L., Hasler, B. (2006) Modelling cost-efficient reductions of nutrient loads to the Baltic Sea, NERI technical report no 592. | | | yes |

| | | | | | |
|---|---|---|---|---|---|
| 0 | | Schultz-Zehden, A., Matczak, M. (2012) Knowledge gaps conclusions, Submariner Compendium. An assessment of innovative and sustainable uses of Baltic marine resources. Maritime institute in Gdansk. | | | yes |
| 0 | | Skarbö, R.A., Ehlers, S., Aesöy, V. (2015) Emission reduction technology and cost efficiency for ships operating on the northern sea route: a case study, conference paper. | | | yes |
| 0 | | Smart, J.C.R., Hasler, B., Czajkowski, M., Smedberg, E., Fonnesbech-Wulff, A., Termansen, M. (2011) A translog approach for estimating the costs of improving waste water treatment in catchments draining into the Baltic Sea, conference abstract. | | | yes |
| 1 | 0 | Stadmark, J., Conley, D.J. (2011) Mussel farming as a nutrient reduction measure in the Baltic Sea: Consideration of nutrient biogeochemical cycles,Marine Pollution Bulletin 62(7):1385. | | yes | |
| 0 | | Strand, J. A., & Weisner, S. E. B. (2013) Effects of wetland construction on nitrogen transport and species richness in the agricultural landscape—Experiences from Sweden, Ecological Engineering 56: 14-25. | | | yes |
| 1 | 0 | Sumelius, J. (1994) Controlling nonpoint source pollution of nitrogen from agriculture through economic instruments in Finland, report: Research publications 74, Agricultural economics research institute, Finland. | | | yes |
| 0 | | Suutari, M. (2020) Potential of filamentous macroalgae and sessile invertebrates for bioremediation and valorization in the northern Baltic Sea, PhD thesis. | | | yes |
| 0 | | Swain, R.B. (2017) Environmental challenges in the Baltic Region: a perspective from economics. | | | yes |

| | | | | | |
|---|---|---|---|---|---|
| 1 | 0 | Szoege, H. M., & Sobolewska, A.  (2004)  Cost effectiveness of some environmental projects in agriculture in Poland and the EU countries, Electronic Journal of Polish Agricultural Universities 7(1). | | | yes |
| 1 | 0 | Söderqvist, T. (2002) Constructed wetlands as nitrogen sinks in southern Sweden: an empirical analysis of cost determinants, Ecological engineering 19(2):161-173. | | | yes |
| 0 | | Timmermann, K., Maar, M., Bolding, K., Larsen, J., Windolf, J., Nielsen, P., Petersen, JK (2019) Aquaculture Environment Interactions,11 (191-204). | | | yes |
| 0 | | Torn, K., Martin, G., Suursaar, Ü. (2016) Beach wrack macrovegetation index for assessing coastal phytobenthic biodiversity, Proceedings of the Estonian Academy of Sciences 65(1): 78-87. | | yes | |
| 0 | | Tredanari, A. (2011) The effect of buffer strip width on cost efficiency, Master's thesis. | | | yes |
| 0 | | Trepel, M., & Palmeri, L.  (2002)  Quantifying nitrogen retention in surface flow wetlands for environmental planning at the landscape-scale, Ecological Engineering 19(2):127-140. | | | yes |
| 1 | 0 | Trepel, M., & Palmeri, L.  (2010) Assessing the cost-effectiveness of the water purification function of wetlands for environmental planning, Ecological complexity 7(3):320-326. | | | yes |
| 1 | 0 | Turner, R. K., Georgiou, S., Gren, I.-M., Wulff, F., Barrett, S., Söderqvist, T., . . . Markowska, A.  (1999)  Managing nutrient fluxes and pollution in the Baltic: an interdisciplinary simulation study, Ecological Economics 30(2):333-352. | | | yes |

| | | | | | |
|---|---|---|---|---|---|
| 0 | | Tynkkynen, N. (2015) Baltic Sea Environment, Knowledge and the Politics of Scale, Journal of Environmental Policy & Planning 17(2): 201-216. | | | yes |
| 0 | | Tynkkynen, N., Schönach, P., Pihlajamäki, M., & Nechiporuk, D. (2014) The Governance of the Mitigation of the Baltic Sea Eutrophication: Exploring the Challenges of the Formal Governing System, AMBIO 43(1): 105-114. | | | yes |
| 0 | | Ulvi, T., Visuri, M., Hellsten, S. (2007) Proceedings of the European Symposium of Spatial Planning Approaches towards Sustainable Riven Basin Management, Reports of Finnish environment institute 12, 2007. | | | yes |
| 0 | | Unknown (2007) 10th International Symposium on Practical Design of Ships and other Floating Structures, PRADS 2007, Volume 2. | | yes | |
| 0 | | Unknown (2007) 10th International Symposium on Practical Design of Ships and other Floating Structures, PRADS 2007, Volume 1. | | yes | |
| 0 | | Wojciechowska, E., Pietrzak, S., Matej-Łukowicz, K., Nawrot, N., Zima, P., Kalinowska, D., . . . Dzierzbicka-Głowacka, L. (2019) Nutrient loss from three small-size watersheds in the southern Baltic Sea in relation to agricultural practices and policy, Journal of Environmental Management 252. | | | yes |
| 1 | 1 | Wulff, F., Humborg, C., Andersen, H. E., Blicher-Mathiesen, G., Czajkowski, M., Elofsson, K., . . . Żylicz, T. (2014) Reduction of Baltic Sea Nutrient Inputs and Allocation of Abatement Costs Within the Baltic Sea Catchment, AMBIO 43(1): 11-25. | | | yes |

| | | | | | |
|---|---|---|---|---|---|
| 0 | | Vuorinen, P.J., Saulamo, K., Lecklin, T., Rahikainen, M., Koivisto, P., Keinänen, M. (2017) Baseline concentrations of biliary PAH matabolites in perch (Perca fluviatilis) in the open Gulf of Finland and in two coastal areas, Journal of Marine Systems 171: 134-140. | | yes | |
| 0 | | Zalewski, M. (2014) Ecohydrology, biotechnology and engineering for cost efficiency in reaching the sustainability of biogeosphere, Ecohydrology & Hydrobiology 14(1): 14-20. | | | yes |
| 0 | | Zanou, B., Kontogianni, A., & Skourtos, M. (2004) Principles for the application of the cost-effectiveness analysis in water quality sector, International Journal of Water 2(4):297-311. | | | yes |
| 0 | | Zanou, B. (2006) Decrease of non-point water pollution: A practical algorithm for the user-friendly presentation of the cost effectiveness comparison of management measures, E-Water. | | | yes |
| 1 | 0 | Zylicz, T. (1993) The ecological economics of the Baltic Sea, European review, 1(4): 329-335. | | yes | yes |
| 1 | 0 | Zylicz, T. (2003) Instruments for water management at the drainage basin scale, Ecological Economics, 47(1): 43-51. | | | yes |

Table S2 Abatement measures used in different studies*

| | Gren et al. (1997) | Elofsson (1999) | Elofsson (2003) | Schou et al. (2006) | Gren et al. (2008) | Gren (2008a) | Gren (2008b) |
|---|---|---|---|---|---|---|---|
| Catalysts in cars | x | | | | | | |
| Catalysts in ships | x | | | | x | x | x |
| Catalysts in trucks | | | | | x | x | x |
| Catalysts in power plants | x | | | x | x | x | x |
| Improved wastewater treatment | x | | x | x | x | x | x |
| Private sewers | | | | | x | x | x |
| P-free detergents | | | | | x | x | x |
| Construction of sedimentation ponds | | | | | | | |
| Mussel farming | | | | | | x | |
| Wetlands | x | | | x | x | x | x |
| Buffer strips | | x | | | x | x | x |
| Change in the spreading time of manure | x | x | x | | x | x | x |
| Cultivation of catch crops | x | x | x | x | x | x | x |
| Energy forestry | x | x | x | | x | x | x |
| Fallow with cover crop | | x | x | | | | |
| Fertiliser reduction | x | x | x | x | x | x | x |
| Grasslands | x | x | x | | x | x | x |
| Reduction of cattle | x | x | x | x | x | x | x |
| Reduction of pigs | x | x | x | x | x | x | x |
| Reduction of poultry | x | x | x | | x | x | x |
| Soil drainage | | | | | | | |
| Winter crops | | x | x | | | | |

* Note that Ollikainen and Hautakangas (2001) is not included in the table as they do not define which measures that are included in their model.

| | Gren (2008c) | Gren et al. (2009) | Bryhn (2009) | Elofsson (2010b) | Elofsson (2010c) | Gren & Destouni (2012) | Gren et al. (2013) | Lindqvist & Gren (2013) | Lindqvist et al. (2013) |
|---|---|---|---|---|---|---|---|---|---|
| Catalysts in cars | x | | | | | | | | |
| Catalysts in ships | x | x | | x | x | | x | x | x |
| Catalysts in trucks | | x | | x | x | | x | x | x |
| Catalysts in power plants | x | x | | x | x | | x | x | x |
| Improved wastewater treatment | x | x | x | x | x | x | x | x | x |
| Private sewers | | x | x | x | x | x | x | x | x |
| P-free detergents | | x | x | x | x | x | x | x | x |
| Construction of sedimentation ponds | | | x | | | | | | |
| Mussel farming | | x | x | | | | | | |
| Wetlands | x | x | x | x | x | x | x | x | x |
| Buffer strips | | x | x | x | x | | x | x | x |
| Change in the spreading time of manure | x | x | | x | x | x | x | x | x |
| Cultivation of catch crops | x | x | | x | x | x | x | x | x |
| Energy forestry | x | x | | x | x | x | x | x | x |
| Fallow with cover crop | | | | | | | | | |
| Fertiliser reduction | x | x | | x | x | x | x | x | x |
| Grasslands | x | x | | x | x | x | x | x | x |
| Reduction of cattle | x | x | x | x | x | x | x | x | x |
| Reduction of pigs | x | x | x | x | x | x | x | x | x |
| Reduction of poultry | x | x | | x | x | x | x | x | x |
| Soil drainage | | | x | | | | | | |
| Winter crops | | | | | | | | | |

| | Elofsson (2014) | Hautakangas et al. (2014) | Gren & Säll (2015)* | Gren (2017) | Gren et al. (2018) | Gren & Ang (2019)* | Häggmark Svensson & Elofsson (2019) | Czajkowski et al. (2019) |
|---|---|---|---|---|---|---|---|---|
| Catalysts in cars | x | | x | | | x | | |
| Catalysts in ships | x | | x | x | x | x | | |
| Catalysts in trucks | | | | x | x | | | |
| Catalysts in power plants | | | x | x | x | x | | |
| Improved wastewater treatment | x | x | x | x | x | x | x | |
| Private sewers | | | | x | x | | x | |
| P-free detergents | | | | x | x | | | |
| Construction of sedimentation ponds | | | | | | | | |
| Mussel farming | | | | | x | | | |
| Wetlands | x | | x | x | x | x | x | |
| Buffer strips | | | | x | x | | | |
| Change in the spreading time of manure | x | | | x | x | | x | |
| Cultivation of catch crops | x | | x | x | x | x | x | |
| Energy forestry | x | | x | x | x | x | x | |
| Fallow with cover crop | | | | | | | | |
| Fertiliser reduction | x | | x | x | x | x | x | x |
| Grasslands | x | | x | x | x | x | x | |
| Reduction of cattle | x | | x | x | x | x | x | x |
| Reduction of pigs | x | | x | x | x | x | x | x |
| Reduction of poultry | x | | x | x | x | x | x | x |
| Soil drainage | | | | | | | | |
| Winter crops | | | | | | | | |

* Additional measures focusing on fossil fuels: decreased use of fossil fuels, replacement of fossil fuel for heating by wind, solar power and bioenergy.

| | Hasler et al. (2014) | Wulff et al. (2014) | Ahlvik et al. (2014) | BalticSTERN & SwAM (2013)[1] | Hyytiäinen et al. (2014)[2] | Hyytiäinen et al. (2015) | Hyytiäinen and Ahlvik (2015) | Nainggolan et al. (2018) |
|---|---|---|---|---|---|---|---|---|
| Catalysts in ships | | | | | | | | |
| Catalysts in trucks | | | | | | | | |
| Catalysts in power plants | | | | | | | | |
| Improved wastewater treatment | x | x | x | x | x | x | x | x |
| Private sewers | | | | | | | | |
| P-free detergents | | | x | x | | x | x | |
| Construction of sedimentation ponds | | | x | x | | x | x | |
| Mussel farming | | | | | | | | |
| Wetlands | x | x | x | x | x | x | x | x |
| Buffer strips | | | | | | | | |
| Change in the spreading time of manure | | | | | | | | |
| Cultivation of catch crops | x | x | x | x | x | x | x | x |
| Energy forestry | | | | | | | | |
| Fallow with cover crop | | | | | | | | |
| Fertiliser reduction | x | x | x | x | x | x | x | x |
| Grasslands | | | | | | | | |
| Reduction of cattle | x | x | x | x | x | x | x | x |
| Reduction of pigs | x | x | x | x | x | x | x | x |
| Reduction of poultry | | | x | x | | x | x | |
| Soil drainage | | | | | | | | |
| Winter crops | | | | | | | | |

[1] Measures reported here are only from the main MTT calculations even if BALTCOST calculations also are made.
[2] Measures reported here are only from the main BALTCOST calculations even if MTT calculations also are made.

Table S3 Cost-effectiveness studies and their cost estimates, all costs are annual unless stated otherwise (N = nitrogen, P = phosphorus)

¤ Costs are stated in Swedish crowns (SEK) in the paper and no exchange rate is mentioned. We have therefore converted all figures in SEK to EUR using exchange rates from the National Bank of Sweden: https://www.riksbank.se/sv/statistik/sok-rantor--valutakurser/

| | Gren et al. (1997) | Elofsson (1999) | Ollikainen and Honkatukia (2001) | Elofsson (2003) |
|---|---|---|---|---|
| **Model** | Static | Static | Static | Static |
| **Baseline load** | N:730 000 tonnes<br>P: 37 000 tonnes | N from arable land: 255 224 tonnes<br><br>Total N load:<br>1 313 800 tonnes | N: 864 327 tonnes<br>P: 67 849 tonnes | N from arable land: 256 991 tonnes<br>N from coastal point sources : 75 281 tonnes<br><br>P from arable land: 7159 tonnes<br>P from coastal point sources: 7139 tonnes |
| **Target** | 1.Overall reduction of N (365 000 tonnes per year) and P (18 500 tonnes per year) by 50%<br>2. Proportional reduction, all countries reduce N and P by 50% | 1.Overall reduction of N by 50% (127 000 tonnes)<br><br>2. Proportional reduction, all countries reduce N by 50% | 1.Overall reduction of N (432 164 tonnes per year) and P (33 925 tonnes) by 50%<br>2. Proportional reduction, all countries reduce N and P by 50% | Reduction of N and P by 50%, different levels of certainty |
| **Cost** | 1.1.4 billion EUR (N),0.35 billion EUR (P)¤<br><br>2. 6 billion EUR (N), 1.6 billion EUR (P) ¤ | 1. 1.3 billion EUR¤<br><br>2. 2.1 billion EUR¤ | 1.15.8 billion EUR (N), 0.8 billion EUR (P)<br>2. 105 billion EUR (N), 7.7 billion EUR (P) | Simultaneous reduction:1.12 billion EUR¤<br><br>80% certainty of reaching the target: 1.5 billion EUR; 95% certainty of reaching the target: 2 billion EUR¤ |

| | Schou et al. (2006) | Gren et al. (2008) | Gren (2008a) | Gren (2008b) | Gren (2008c) |
|---|---|---|---|---|---|
| **Model** | Static | Static | Static | Model and data from Gren et al. (2008). Adds one abatement measure. | Model and data from Gren et al. (2008). |
| **Baseline loads** | N:745 000 tonnes | N: 824 000 tonnes<br>P: 38 900 tonnes | N: 952 000 tonnes | N: 824 000 tonnes<br>P: 38 900 tonnes | N: 824 000 tonnes<br>P: 38 900 tonnes |
| **Target** | N reduction by about 20% (160 000 tonnes per year) | 1. Overall N reductions ranging between 0 and 50 %, overall P reductions ranging from 0 to 70%.<br><br>2. N and P reductions up to 50% to marine basins | Main results are based on N reductions by 20%. Overall and national targets are compared for different values of the correlation coefficient between upstream emission and wetland abatement. | 1.2007 BSAP country-specific targets,<br><br>2. Cost-effective solution where overall reductions are equivalent to 2007 BSAP targets (25% N red., 54% P red. | As in Gren et al. (2008) but also:<br>1. 2007 BSAP basin targets (30% N, 69% P red.)<br><br>2. 2007 BSAP country-specific targets per basin (21% N red., 48% P red.)<br><br>3. Overall reductions equivalent to 2007 BSAP country targets (21% N red., 48% P red.) |
| **Cost** | 0.8 billion EUR | 1. 2.9 billion EUR (50% N red.), 1.8 billion EUR (50% P red.)¤<br><br>2. About 4.5 billion EUR for simultaneous N and P reductions by 50% to marine basins. ¤<br>(Note: no exact figure is mentioned in the paper, only a graph is shown.) | Overall reductions: 0.34-0.36 billion EUR¤<br><br>National targets: 0.61-1.43 billion EUR | 1. 5.5 billion I$<br><br>2. 4 billion I$ | 1. 4.68 billion EUR<br><br>2. 2.56 billion EUR<br><br>3. 1.62 billion EUR |

| | Bryhn (2009) | Gren et al. (2009) | Elofsson (2010c)[†] | Gren & Destouni (2012) | Gren et al. (2013) |
|---|---|---|---|---|---|
| **Model** | No model used | Gren et al. (2008) model with mussel farming | Static model. Data from Gren et al. (2008) | Gren et al. (2008) model | Dynamic model. Data on abatement measures from Gren et al. (2008). |
| **Baseline loads** | Uses Seechi depths not annual loads for calculations. Pre-1960s Secchi depths: 8 m | Not mentioned but data are taken from Gren et al. (2008). | 1997-2003 loads from HELCOM:<br>N: 736 714 tonnes<br>P: 36 310 tonnes. | 1. N: 736 000 tonnes, P: 36 300 tonnes;<br>2. N: 685 000 tonnes, P: 35 000 tonnes;<br>3. N: 688 000 tonnes, P: 31 400 tonnes | N: 634 800 tonnes<br>P: 33 800 tonnes |
| **Target** | Restore the Secchi depth (from 5.5 to 8 m) and cyanobacterial blooms to their pre-1960s level.<br><br>1. 6650 tonnes reduction of P,<br>2. 10200 tonnes reduction of P. | 1. Overall reduction ranging between 0 and 60 % for N, overall reduction ranging from 0 to 70% for P.<br><br>2. 2007 BSAP (overall N reduction by 25%, overall P reduction by 66%) with and without mussel farming | 1. Cost-effective 2007 BSAP basin targets (143526 tonnes N red., 13213 tonnes P red.)<br><br>2. 2007 BSAP country-and-basin targets (163 616 tonnes N red., 13 256 tonnes P red.) | Targets based on 2007 BSAP overall basin targets.<br>N: 601 000 tonnes,<br>P: 21 100 tonnes | 2007 BSAP nutrient pool targets per basin achieved by 2082.<br><br>1. Separate reduction of N (197000 tonnes N red.) and P (230000 tonnes P red.)<br><br>2. Simultaneous reduction of N and P, targets achieved by 2082 |
| **Cost** | 1. 0.21 billion EUR<br><br>2. 0.43 billion EUR | 1. **Without mussel farming.** Separate 50% reductions: 2.3 billion EUR (N), 1.6 billion EUR (P). Simultaneous reduction: 3.2 billion EUR. **With mussel farming.** Separate 50% reductions: 2.16-2.23 billion EUR (N), 1.55-1.59 billion EUR (P). Simultaneous reduction: 3.0-3.1 billion EUR.<br><br>2. **Without mussel farming**: 3.15 billion EUR. Mussel farming can give cost savings ranging between 20 and 138 million EUR. | 1. 3.8 billion EUR (after nutrient load trading)<br><br>2. 4.53 billion EUR | 1. 2.1 billion EUR<br><br>2. 1.9 billion EUR<br><br>3. 2.3 billion EUR | 1. Total costs: 31 billion EUR (N), 126 billion EUR (P).<br><br>2. Total costs: 132 billion EUR, 1 135 billion SEK, or 1.9 billion EUR per year (discounted average annual cost) |

[†] Elofsson (2010b) contains the same cost estimates as Elofsson (2010c)

| | Lindqvist & Gren (2013) | Lindqvist et al. (2013) | Elofsson (2014) | Hautakangas et al. (2014) | Gren & Säll (2015) |
|---|---|---|---|---|---|
| **Model** | Gren et al. (2013) model with technical change. | Gren et al. (2013) model with a climate change dimension. | Dynamic model. Similar to Gren et al. (2013). Includes international knowledge spillovers. | No cost-effectiveness model is used. | Static. Gren et al. (2008) model with emissions and abatement of GHG |
| **Baseline loads** | N. 657 000 tonnes,<br>P: 33 800 tonnes | N: 657 000 tonnes,<br>P: 33 800 tonnes | N. 635 000 tonnes,<br>P: 33 800 tonnes | Loads from wastewater treatments plants. N: 110 000 tonnes, P: 11 000 tonnes | N: 711 500 tonnes,<br>P: 30 400 tonnes |
| **Target** | 2007 BSAP targets for N and P, appears to be overall basin targets. Different scenarios with respect to technical change are analysed. Targets should be achieved by 2100 and then sustained for another 70 years. | 2007 BSAP targets for N and P, appears to be overall basin targets. Four climate change scenarios are analysed while demographic and agricultural changes are taken into account. Targets should be achieved by 2100 and then sustained for another 70 years. | 2007 BSAP nutrient pool targets for sea basins for N and P as in Gren et al. (2013). The analysis mainly focuses on how knowledge spillovers affect abatement costs. | Wastewater treatment should abate 70-90% (44 000-83 000 tonnes more than at present) of nitrogen emissions and 80-95% (5700-9400 tonnes more than at present) of phosphorous emissions. The target is met when every plant is operating at the required reduction level. | Targets based on 2013 BSAP: reduction of N by 23%, reduction of P by 60%, reduction of CO2e by 10%. Appears to be overall reductions that are modelled.<br><br>Separate vs. simultaneous reductions. |
| **Cost** | Costs are only shown in a graph, no exact figures are given. Technical change reduces costs. For example, total abatement costs are reduced by 44% for a learning rate of 12% for each doubling of abatement. | Total abatement costs when climate change, demographic and agricultural changes are taken into account: 500-922 billion EUR¤<br><br>Abatement costs decrease with the severity of climate change and reductions in population but increase with agricultural changes (increased animal protein production). | Total net present costs with 1) no knowledge spillovers: 82.4 billion EUR¤ 2) domestic knowledge spillovers: 68 billion EUR, 3) international knowledge spillovers 64-67 billion EUR. Note: no exact figure is mentioned in the paper, only a graph is shown. | The total costs to meet the 70% abatement level for N: 310 million EUR. Increasing the abatement level to 90% costs 670 million EUR. The total costs to meet the 80% abatement level for P: 95 million EUR. Increasing the abatement level to 90% costs 150 million EUR. | Separate reductions: 1.1 billion EUR (N), 4.0 billion EUR (P), 2.6 billion EUR (CO2e).<br><br>Simultaneous reduction: 6.4 billion EUR. |

| | Gren (2017) | Gren et al. (2018) | Gren & Ang (2019) | Häggmark Svensson & Elofsson (2019) | Czajkowski et al. (2019) |
|---|---|---|---|---|---|
| **Model** | Gren et al. (2013) model with climate change and technical change as conditions of uncertainty. | Static. Data from Gren & Säll (2015) and Gren et al. (2009). | Static model that takes greenhouse gases and the possibility of stacking abatement into account. | Elofsson (2010b) model | Static |
| **Baseline loads** | N: 824 000 tonnes,<br><br>P: 38 900 tonnes. | N: 814 000 tonnes,<br><br>P: 34 810 tonnes | N: 711 500 tonnes,<br><br>P: 30 400 tonnes | Baseline years 1992-1996.<br><br>N: 889 495 tonnes. | N: 340 000 tonnes |
| **Target** | Overall N reductions by 15% and P reductions by 50%, in relation to the initial nutrient pools (N: 2 567 000 tonnes, P: 558 000 tonnes). A time period of 60 years is applied. | Targets based on the 2013 BSAP. N reduction by 11% (89 650 tonnes) and P reduction by 42% (14 374 tonnes). Overall reduction is compared to the country targets set by the BSAP. | Targets based on the 2013 BSAP targets. Reduction of the nitrogen load by 28%, reduction of the phosphorus load by 51%, 10% reduction of $CO_2$ emissions. | N reduction due to water quality policies: 145 535 tonnes | Reduction between 5 and 50% of maximum potential reduction from current N loads in each region (from Baltic Sea level down to grid square level) |
| **Cost** | Average annual costs without climate change and technical change: 1.3 billion EUR.¤ Costs can decrease by 50% or increase by 125% depending on impacts of climate change, technical change, and the level of reliability of achieving the targets. | **Cost-effective solution** Total abatement costs without mussel farming: 3.3 billion EUR. Total costs with mussel farming: 3.0 Billion EUR.<br><br>**BSAP country targets** Total abatement costs without mussel farming: 4.9 billion EUR. Total costs with mussel farming: 4.6 billion EUR. | **With stacking** Total abatement cost: 4.699 billion EUR. Total cost of nitrogen and phosphorus reduction only : 3.302 billion Euro<br><br>**Without stacking** Total abatement cost: 7.026 billion EUR when all pollutants are regulated by price mechanisms. | Cost of actual reductions: 2.093 billion EUR.<br><br>Least-cost solution for the same overall reduction: 250 million EUR | N reduction by 50% (141 100 tonnes): 89-169 million EUR |

| | Wulff et al. (2014) | Hasler et al. (2014) | Ahlvik et al. (2014) | BalticSTERN & SwAM (2013) | Hyytiäinen et al. (2014) |
|---|---|---|---|---|---|
| **Model** | BALTCOST | BALTCOST | MTT | MTT and BALTCOST | MTT and BALTCOST |
| **Baseline loads** | Averages for the period 1994-2006 taken from HELCOM PLC-5. No numbers given in the paper. | Averages for the period 1997-2003 taken from HELCOM's 2013 revision of targets. No numbers given in the paper but these are the official HELCOM loads:<br><br>N: 910 343 tonnes<br>P: 36 893 tonnes | Averages for the period 2004-2008:<br><br>N: 706 704 tonnes<br>P: 32 720 tonnes | Averages for the period 2004-2008:<br><br>N: 706 705 tonnes<br>P: 32 719 tonnes | Original averages for the period 1997-2003 from HELCOM:<br>N: 736 720 tonnes<br>P: 36 310 tonnes<br><br>Averages for the period 2004-2008:<br>N: 706 702 tonnes<br>P: 32 720 tonnes |
| **Target** | 2007 BSAP basin targets (133 120 tonnes N red., 12 040 tonnes P red.) | 2013 BSAP basin targets, simultaneous reduction of N and P (118 131 tonnes N red., 15 174 tonnes P red.)<br><br>Reduction achieved: 214 131 tonnes N red., 12 503 tonnes P red. | Good environmental status based on the 2007 BSAP targets (93 819 tonnes N red., 12 244 tonnes P red.) | 1. 2007 BSAP country-specific targets per basin (115 530 tonnes N red., 11 818 tonnes P red.)<br><br>2. 2007 BSAP basin targets (108 850 tonnes N red., 11 409 tonnes P red.) | 1. 2007 BSAP basin targets (N: 601 710 tonnes, P: 21 060 tonnes)<br>a. 1997-2003 loads as baseline<br>b. 2004-2008 loads as baseline<br><br>2. 2007 BSAP country-specific targets per basin with 2004-2008 loads as baseline |
| **Cost** | 4.7 billion EUR | 4.2 billion EUR | 1.5 billion EUR per year for 40 years | MTT results:<br>1. 2.8 billion EUR<br>2. 2.3 billion EUR<br><br>BALTCOST results:<br>2. 1.4 billion EUR | BALTCOST results:<br>1a. 4.7 billion EUR<br>1b. 1.4 billion EUR<br><br>MTT results:<br>1b. 2.3 billion EUR per year for 40 years<br>2. 2.8 billion EUR per year for 40 years |

| | Hyytiäinen et al. (2015) | Hyytiäinen and Ahlvik (2015) | Nainggolan et al. (2018) |
|---|---|---|---|
| **Model** | MTT | MTT | BALTCOST |
| **Baseline loads** | Averages for the period 2004-2008:<br><br>N: 706 710 tonnes<br>P: 32 719 tonnes | Averages for the period 2004-2008:<br><br>N: 706 704 tonnes<br>P: 32 719 tonnes | Unclear but most likely the same as in Hasler et al. (2014), i.e. averages for the period 1997-2003 taken from HELCOM's 2013 revision of targets. |
| **Target** | 1. 2007 BSAP country-specific targets per basin (N: 591 182 tonnes, P: 20901 tonnes)<br><br>2. 2007 BSAP basin targets cost-effectively (N: 597 861 tonnes, P: 21 310 tonnes)<br><br>3. 2007 BSAP state of the sea targets (N: 612 891 tonnes, P: 20 475 tonnes)<br><br>4. optimised nutrient abatement in the Baltic Sea<br><br>5. optimised nutrient abatement guaranteeing positive net benefits for each littoral country | 1.2013 BSAP country-specific targets per basin (N: 610 143 tonnes, P: 20 427 tonnes)<br><br>2. 2013 BSAP basin targets (N: 606 913 tonnes, P: 19 630 tonnes)<br><br>3. 2013 BSAP flexible basin targets | 1. 2013 BSAP basin targets (118 131 tonnes N red., 15 174 tonnes P red.)<br><br>2. 2013 BSAP basin targets and GHG reduction |
| **Cost** | 1.2.8 billion EUR per year for 40 years<br><br>2. 2.3 billion EUR per year for 40 years<br><br>3. 1.5 billion EUR per year for 40 years<br><br>4. 0.6 billion EUR per year for 40 years<br><br>5. 0.5 billion EUR per year for 40 years | 1. 2 billion EUR per year for 30 years<br><br>2. 1.7 billion EUR per year for 30 years<br><br>3. 1.5 billion EUR per year for 30 years | 1. 4.2 billion EUR (7.1 million tonnes reduction of GHG),<br><br>2. 6.3 billion euros (18.6 million tonnes reduction of GHG) |

## References


Ahlvik, L., and Y. Pavlova. 2013. A strategic analysis of eutrophication abatement in the Baltic Sea. *Environmental and Resource Economics* 56: 353-378.

BalticSTERN & SwAM. 2013. The Baltic Sea - Our common treasure. Economics of saving the sea. BalticSTERN and Swedish Agency for Marine and Water Management, Report 2013:4, Gothenburg.

Bryhn, A.C. 2009. Sustainable phosphorus loadings from effective and cost-effective phosphorus management around the Baltic Sea. *PloS one* 4:e5417.

Czajkowski, M., H.E. Andersen, G. Blicher-Mathiasen, W. Budzinski, K. Elofsson, J. Hagemejer, B. Hasler, C. Humborg et al. 2019. Increasing the cost-effectiveness of water quality improvements through pollution abatement target-setting at different spatial scales. Discussion Papers in Environmental and One Health Economics, University of Glasgow**,** Paper Number 2019-05.

Elofsson, K. 1999. Cost Effective Reductions in the Agricultural Load of Nitrogen to the Baltic Sea. In *Topics in Environmental Economics, Economy & Environment vol. 17*. ed. M. Boman, R. Brännlund and B. Kriström, 121-154. Dordrecht: Springer.

Elofsson, K. 2003. Cost-Effective Reductions of Stochastic Agricultural Loads to the Baltic Sea. *Ecological Economics* 47: 13-31.

Elofsson, K. 2010b. Baltic-Wide and Swedish Nutrient Reduction Targets. An evaluation of cost-effective strategies. Ministry of Finance, Report to the Expert Group for Environmental Studies 2010:2. Stockholm.

Elofsson, K. 2010c. Cost-effectiveness of the Baltic Sea Action Plan. *Marine Policy* 34: 1043-1050.

Elofsson, K. 2014. International knowledge diffusion and its impact on the cost-effective clean-up of the Baltic Sea. Department of Economics, Swedish University of Agricultural Sciences, Working paper 2014:06.

Gren, I.-M. 2008a. Adaptation and mitigation strategies for controlling stochastic water pollution: An application to the Baltic Sea. *Ecological Economics* 66: 337-347.

Gren, I.-M. 2008b. Cost effectiveness and fairness of the HELCOM Baltic Sea Action Plan against eutrophication. *Vatten* 64: 273-281.

Gren, I.-M. 2008c. Costs and benefits from nutrient reductions to the Baltic Sea. Swedish Environmental Protection Agency, Report 5877, Stockholm.

Gren, I.-M. 2017. Costs of Nutrient Management with Technological Development and Climate Change. In *Environmental Challenges in the Baltic Region: A Perspective from Economics* ed. R. Bali Swain. Cham: Springer.

Gren, I.-M. and F. Ang. 2019. Stacking of abatement credits for cost-effective achievement of climate and water targets. *Ecological Economics* 164: 106375.

Gren, I.-M. and G. Destouni. 2012. Does divergence of nutrient load measurements matter for successful mitigation of marine eutrophication? *Ambio* 41: 151-160.

Gren, I.-M. and S. Säll. 2015. Cost-effective and green-house gas management in the Baltic Sea region. *Environmental economics* 6: 80-90.

Gren, I.-M., P. Jannke and K. Elofsson. 1997. Cost-Effective Nutrient Reductions to the Baltic Sea. *Environmental and Resource Economics* 10: 341-362.

Gren, I.-M., Y. Jonzon and M. Lindqvist. 2008. Costs of nutrient reductions to the Baltic Sea: technical report, Department of Economics, Swedish University of Agricultural Sciences, Working Paper 2008:1.

Gren, I.-M., O. Lindahl and M. Lindqvist. 2009. Values of mussel farming for combating eutrophication: An application to the Baltic Sea. *Ecological Engineering* 35: 935-945.

Gren, I.-M., O.P. Savchuk, and T. Jansson. 2013. Cost-Effective Spatial and Dynamic Management of a Eutrophied Baltic Sea. *Marine Resource Economics* 28: 263-284.

Gren, I.-M., S. Säll, A.Z. Aklilu and W. Tirkaso. 2018. Does mussel farming promote cost savings and equity in reaching nutrient targets for the Baltic Sea? *Water* 10(11): 1062.

Hasler, B., J.C.R. Smart, A. Fonnesbech-Wulff, H.E. Andersen, H. Thodsen, G. Blicher Mathiesen, E. Smedberg, C. Göke et al. 2014. Hydro-economic modelling of cost-effective transboundary water quality management in the Baltic Sea. *Water Resources and Economics* 5: 1-23.

Hautakangas, S., M. Ollikainen, K. Aarnos and P. Rantanen. 2014. Nutrient Abatement Potential and Abatement Costs of Waste Water Treatment Plants in the Baltic Sea Region. *Ambio* 43: 352-360.

Hyytiäinen, K. and L. Ahlvik. 2015. Prospects for cost-efficient water protection in the Baltic Sea. *Marine Pollution Bulletin* 90: 188-195.

Hyytiäinen, K., K. Blyh, B. Hasler, L. Ahlvik, H. Ahtiainen, J. Artell and S. Ericsdotter. 2014. Environmental economic research as a tool in the protection of the Baltic Sea - Costs and benefits of redeucing eutrophication. Nordic Council of Ministers, TemaNord 2014:504, Copenhagen.

Hyytiäinen K., L. Ahlvik, H. Ahtiainen, J. Artell, A. Huhtala and K. Dahlbo. 2015. Policy Goals for Improved Water Quality in the Baltic Sea: When do the Benefits Outweigh the Costs? *Environmental & Resource Economics* 61: 217-241.

Häggmark Svensson, T. and K. Elofsson. 2019. The Ex-Post Cost-Effectiveness of Nitrogen Load Reductions From Nine Countries to the Baltic Sea Between 1996 and 2010. *Water Resources Research* 55: 5119-5134.

Lindqvist, M. and I.-M. Gren. 2013. Cost effective nutrient abatment for the Baltic Sea under learning-by-doing induced technical change. Department of Economics, Swedish University of Agricultural Sciences, Working paper 2013:01.

Lindqvist, M., I.-M. Gren and K. Elofsson. 2013. A Study of Climate Change and Cost Effective Mitigation of the Baltic Sea Eutrophication. In *Climate Change - Realities, Impacts Over Ice Cap, Seal Level and Risks* ed. B.R. Singh. IntechOpen.

Nainggolan, D., B. Hasler, H.E. Andersen, S. Gyldenkærne and M. Termansen. 2018. Water Quality Management and Climate Change Mitigation: Cost-effectiveness of Joint Implementation in the Baltic Sea Region. *Ecological Economics* 144: 12-26.

Ollikainen, M. and J. Honkatukia. 2001. Towards Efficient Pollution Control in the Baltic Sea: An Anatomy of Current Failure with Suggestions for Change. *Ambio* 30: 245-253.

Schou, J.S., S.T. Neye, T. Lundhede, L. Martinsen and B. Hasler. 2006. Modelling cost-efficient reductions of nutrient loads to the Baltic Sea - Concept, data and cost functions for the cost minimisation model. National Environmental Research Institute, NERI technical report 592, Copenhagen.

Wulff, F., C. Humborg, H.E. Andersen, G. Blicher-Mathiesen, M. Czajkowski, K. Elofsson, A. Fonnesbech-Wulff, B. Hasler et al. 2014. Reduction of Baltic Sea Nutrient Inputs and Allocation of Abatement Costs Within the Baltic Sea Catchment. *Ambio* 43: 11-25.
